# Genomic Sequencing and Analysis of a Novel Human Cowpox Virus With Mosaic Sequences From North America and Old World Orthopoxvirus

**DOI:** 10.3389/fmicb.2022.868887

**Published:** 2022-05-03

**Authors:** Diana Diaz-Cánova, Ugo L. Moens, Annika Brinkmann, Andreas Nitsche, Malachy Ifeanyi Okeke

**Affiliations:** ^1^Molecular Inflammation Research Group, Department of Medical Biology, UiT - The Arctic University of Norway, Tromsø, Norway; ^2^Highly Pathogenic Viruses, Centre for Biological Threats and Special Pathogens, WHO Reference Laboratory for SARS-CoV-2 and WHO Collaborating Centre for Emerging Infections and Biological Threats, Robert Koch Institute, Berlin, Germany; ^3^Section of Biomedical Sciences, Department of Natural and Environmental Sciences, School of Arts and Sciences, American University of Nigeria, Yola, Nigeria

**Keywords:** poxvirus, phylogenetics, Fennoscandian, Norway, recombination

## Abstract

Orthopoxviruses (OPXVs) not only infect their natural hosts, but some OPXVs can also cause disease in humans. Previously, we partially characterized an OPXV isolated from an 18-year-old male living in Northern Norway. Restriction enzyme analysis and partial genome sequencing characterized this virus as an atypical cowpox virus (CPXV), which we named CPXV-No-H2. In this study, we determined the complete genome sequence of CPXV-No-H2 using Illumina and Nanopore sequencing. Our results showed that the whole CPXV-No-H2 genome is 220,276 base pairs (bp) in length, with inverted terminal repeat regions of approximately 7 kbp, containing 217 predicted genes. Seventeen predicted CPXV-No-H2 proteins were most similar to OPXV proteins from the Old World, including *Ectromelia virus* (ECTV) and *Vaccinia virus*, and North America, *Alaskapox virus* (AKPV). CPXV-No-H2 has a mosaic genome with genes most similar to other OPXV genes, and seven potential recombination events were identified. The phylogenetic analysis showed that CPXV-No-H2 formed a separate clade with the German CPXV isolates CPXV_GerMygEK938_17 and CPXV_Ger2010_MKY, sharing 96.4 and 96.3% nucleotide identity, respectively, and this clade clustered closely with the ECTV-OPXV Abatino clade. CPXV-No-H2 is a mosaic virus that may have arisen out of several recombination events between OPXVs, and its phylogenetic clustering suggests that ECTV-Abatino-like cowpox viruses form a distinct, new clade of cowpox viruses.

## Introduction

Poxvirus is a family of double-stranded DNA viruses that can infect a broad range of hosts, including mammals, birds, reptiles, and insects (International Committee on Taxonomy of Viruses, ICTV^1^). Based on the host, *Poxviridae* is divided into two subfamilies: *Chordopoxvirinae* (poxviruses that infect vertebrates) and *Entomopoxvirinae* (poxviruses that infect insects) (MacLachlan and Dubovi, 2017). Within the subfamily *Chordopoxvirinae*, there is the genus *Orthopoxvirus* (OPXV). They are viruses with large, linear, double-stranded DNA genomes ranging in size from 170 to 250 kbp (Hendrickson et al., 2010).

One of the best-known species among OPXV is *Variola virus* (VARV), the causative agent of smallpox. It was one of the deadliest viruses in human history and was declared to be successfully eradicated in 1980 after a worldwide smallpox vaccination campaign (Strassburg, 1982). Other members of the OPXV genus also cause human diseases, such as *Cowpox virus* (CPXV), *Monkeypox virus* (MPXV), and vaccinia-like virus (Vora et al., 2015; Reynolds et al., 2018; Diaz, 2021; Silva et al., 2021), but those are zoonotic OPXVs. *Variola virus* is the only OPXV that exclusively infected humans in nature. Among the most studied members of OPXVs, *Vaccinia virus* (VACV) is the prototype species. Several VACV strains were used as smallpox vaccines during the world vaccination campaign (Jacobs et al., 2009).

OPXVs can be further divided into New World and Old World OPXVs according to their endemism. The Old World or African-Eurasian OPXV group contains seven species: *VARV*, *VACV*, *MPXV*, *CPXV*, *Camelpox virus* (CMLV), *Ectromelia virus* (ECTV), and *Taterapox virus* (TATV). The New World OPXV group comprises three species that are endemic to North America: *Raccoonpox virus* (RCNV), *Volepox virus* (VPXV), and *Skunkpox virus* (SKPV) (Smithson et al., 2017b).

In recent times, the increased number of reported OPXV infections as well as the emergence of new OPXVs or re-emergence of existing OPXVs has been reported in several countries across the world (Abrahão et al., 2015; Kalthan et al., 2018). Three novel OPXV species have recently been discovered: *Abatino macacapox virus* (OPXV Abatino) in Italy (Cardeti et al., 2017), *Ahkmeta virus* (AKMV) in Georgia (Gao et al., 2018), and *Alaskapox virus* (AKPV) in the United States (Gigante et al., 2019).

The increasing number of OPXV infections in humans could be due to low population immunity against smallpox after the cessation of smallpox vaccination. The vaccinia-like virus infections were reported in different places and host species (Dumbell and Richardson, 1993; Abrahão et al., 2015; Miranda et al., 2017), including humans (Damaso et al., 2007; Megid et al., 2012). In different countries in Africa, human cases of MPXV infections have been reported (Nakoune et al., 2017; Durski et al., 2018; Yinka-Ogunleye et al., 2019; Alakunle et al., 2020); imported MPXV cases were as well reported in Israel, the United Kingdom and Singapore (Vaughan et al., 2018; Erez et al., 2019; Ng et al., 2019). In Europe, cases of cowpox were reported (Tryland et al., 1998; Kalthoff et al., 2014; Ferrier et al., 2021). The distribution of CPXV is in Eurasia (Chantrey et al., 1999; Wolfs et al., 2002; Laakkonen et al., 2006; Vorou et al., 2008; Popova et al., 2017; Diaz, 2021; Ferrier et al., 2021). The natural reservoirs of CPXV are wild rodents (Chantrey et al., 1999; Kinnunen et al., 2011). CPXV has a wide host spectrum, including humans, monkeys, cats, dogs, horses, and farmed llamas (Tryland et al., 1998; Smith et al., 1999; Girling et al., 2011; Prkno et al., 2017; Diaz, 2021). CPXV’s broad range is associated with its large genome, which is the largest genome among OPXVs (Gubser et al., 2004; Carroll et al., 2011). CPXV is polyphyletic (Carroll et al., 2011; Okeke et al., 2014; Franke et al., 2017; Mauldin et al., 2017), and their strains cluster in at least five clades (Mauldin et al., 2017; Jeske et al., 2019). Among them, some clades are more genetically similar to VACV (VACV-like virus) and VARV (VARV-like virus), whereas other CPXV strains appear as single branches and have a mosaic genome that contains genomic parts from different clades (Franke et al., 2017). The genetic heterogeneity inside CPXV could partially be due to recombination processes with other OPXV species or between CPXV clades (Okeke et al., 2012, 2014; Franke et al., 2017).

A poxvirus was isolated from an 18-year-old man living in the county Nordland, Norway (Hansen et al., 2009). Based on the detection of A-type inclusion (ATI) bodies, the sequence and phylogenetic analysis of hemagglutinin (*HA*) gene, cytokine response modifier B (*crmB*) gene, and Chinese hamster ovary host range (*CHOhr*) genes as well as *Hind III* restriction map, this virus was classified as a CPXV and was tentatively named CPXV-No-H2 (Hansen et al., 2009; Okeke et al., 2012). This isolate produces an atypical ATI phenotype, V^+/^, in which the virions are encrusted only in the periphery of ATI (Okeke et al., 2012). The sequencing of two of the three genes (*atip*, *p4c*, and *A27L*) involved in the production of ATI with virions embedded into ATI (V^+^) (Patel and Pickup, 1987; McKelvey et al., 2002; Howard et al., 2010) showed that it has intact *atip* and *p4c* genes. Furthermore, interestingly, the *atip* gene of CPXV-No-H2 closely related to that of ECTV with a bootstrap support of 100%, whereas the *p4c* gene was more diverse compared to the orthologs in other OPXVs (Okeke et al., 2012, 2014).

In this study, we report the whole sequence and genomic characterization of a Norwegian human CPXV isolate, CPXV-No-H2. We annotated the open reading frames, performed recombination analysis, and determined phylogenetic relationships with other OPXV genomes.

## Materials and Methods

### Cell, Virus Culture, and DNA Isolation

The Fennoscandian CPXV No-H2 strain was isolated in 2001 from a human patient from Northern Norway (Hansen et al., 2009; Okeke et al., 2012). CPXV-No-H2 was cultured on a monolayer of Vero cells (ATCC No. CCL-81) in 175-cm^2^ flasks (NUNC Sweden) as previously described (Okeke et al., 2012). Viral DNA was extracted from semi-purified virions using QIAGEN Genomic-tip 100/G and QIAGEN Genomic DNA Buffer Set, following the manufacturer’s instructions (Qiagen, Hilden, Germany). DNA concentration was measured using NanoDrop 2000 spectrophotometer (Thermo Fischer Scientific™, Waltham, MA, United States).

### Whole-Genome Sequencing

The genome of CPXV-No-H2 was sequenced using Illumina and Oxford Nanopore Technologies (ONT; Oxford, United Kingdom), respectively. The preparation of sequencing libraries and next-generation sequencing with Illumina was performed at the Norwegian Sequencing Centre, Oslo. ThruPLEX DNA-Seq kit with an input DNA of 50 ng was used for the library preparation. Whole-genome sequencing was performed on an Illumina MiSeq instrument (Illumina Inc., San Diego, CA, United States) using MiSeq Reagent v3 (600 cycles), producing 2×300-bp paired-end reads. For nanopore sequencing, sequencing libraries were prepared using the Ligation Sequencing Kit SQK-LSK109 (ONT, Oxford, United Kingdom) and native barcoding expansion kit EXP-NBD104 and EXP-NBD114 (ONT). Up to 14 samples were multiplexed on R9.4 flow cells (FLO-MIN106). The run was performed on GridION X5 (Oxford, United Kingdom) using MinKNOW v20.10.6. Library preparation and nanopore sequencing were performed at the Genomics Support Centre Tromsø at UiT-The Arctic University of Norway.

### Genome Assembly

Raw sequencing data from Illumina MiSeq were evaluated for their quality using FastQC software v0.11.8 (Andrews, 2010). Adapter removal and quality filtering were conducted using Trimmomatic v0.39 (Parameters: ILLUMINACLIP:TruSeq3-PE-2.fa:2:30:10 LEADING:3 TRAILING:3 SLIDINGWINDOW:4:20 MINLEN:36) (Bolger et al., 2014). In order to remove reads corresponding to host cells, filtered reads were mapped against *Chlorocebus sabaeus* (GCF_000409795.2) using FastQ Screen v0.14.1 (Wingett and Andrews, 2018) with BWA v.0.7.17 (Li and Durbin, 2009). The remaining reads were used in the genome assembly. Raw nanopore data (fast5 files) were base called using Guppy 4.2.3 in MinKNOW 20.10.6, with a qscore of 7 as filter, to produce Fastq formatted sequence files. Fastq sequences were demultiplexed using Guppy 4.2.3—likewise with barcode removal. Host sequences were filtered out using FastQ Screen v0.14.1 (Wingett and Andrews, 2018) with BWA v.0.7.17 (Li and Durbin, 2009) as described above. SPAdes v3.15.3 (Bankevich et al., 2012) was used to combine the ONT long reads and the Illumina reads to produce a hybrid assembly (with nanopore option and default parameters). Contigs were screened using BLAST^2^ to remove host contamination. In order to assemble the complete genome, the Illumina reads were mapped to the contigs using Geneious mapper implemented in Geneious Prime 2020.2.4 (Biomatters, Inc., Newark, NJ, United States). Then, the extended contigs were merged into one by Geneious assembler in Geneious Prime 2020.2.4.

### Genome Annotation

The assembled genome was annotated using Genome Annotation Transfer Utility (GATU) software from the Viral Bioinformatics Resource Centre (Tcherepanov et al., 2006). ECTV Moscow strain (ECTV_Mos), CPXV Brighton Red strain (CPXV_Br), and VACV Copenhagen strain (VACV_Cop) were used as reference genomes. These reference sequences were retrieved from the Viral Orthologous Clusters (VOCs) database (Ehlers et al., 2002). The GATU parameters included open reading frames (ORFs) longer than 30 amino acids, with a maximum overlap of 25%. Gene annotations from the reference genomes were transferred to the CPXV-No-H2 genome when the level of similarity was ≥80%. The putative coding sequences (CDS) with low similarity to the reference genes were subjected to a BLASTp analysis against the proteins belonging to the *Poxviridae* family from the NCBI database. Putative CDS with high similarity to other poxviruses were annotated. Similarly, the unassigned ORFs were investigated using BLASTp searches to find orthologous genes. In cases where more than one CDS were found in the same genomic region, the CDS with the highest similarity was selected. Geneious Prime 2020.2.4 was used to visualize, edit, and correct the annotations, if needed.

### Phylogenetic Analysis

For phylogenetic analysis, 75 OPXV genomes were retrieved from the VOCs database (Ehlers et al., 2002), except for CPXV_GerMygEK938_17, which was retrieved from GenBank. The OPXV genomes used in this study are listed in Supplementary Table 1. The alignments of (1) the genomes, excluding the inverted terminal repeats (ITRs; called core genome), (2) the genomic region from the first gene until the last gene (referred to as the whole genome), and (3) the orthologous genes of the 76 OPXVs (including CPXV-No-H2) were performed using MAFFT v1.4.0 (with default parameters; Katoh and Standley, 2013) implemented in Geneious Prime 2020.2.4. The poorly aligned positions were removed from the alignments (1 and 2) with Gblocks 0.91b using default parameters (Talavera and Castresana, 2007). The orthologous genes were identified using OrthoFinder v2.5.2 (Emms and Kelly, 2015). The orthologs (present in ≥95% of the genomes) were aligned as described above and concatenated in Geneious Prime 2020.2.4.

The phylogenetic relationship among these OPXVs was inferred by the maximum likelihood (ML) and Bayesian inference (BI) methods. ML trees were constructed in RAxML v.8.2.12 (Stamatakis, 2014) using the best-fitting nucleotide substitution model and 1,000 bootstrap replicates. The best-fit nucleotide substitution model for the alignment data was selected using the modelTest-NG v.0.1.6 (Darriba et al., 2020). BI analyses were performed using MrBayes v.3.2.7 (Ronquist et al., 2012) under the best-fitting substitution model with the following parameters: 2 million generations, nchains = 4, samplefreq = 500, and burninfrac = 0.25. The phylogenetic trees were visualized using FigTree v1.4.4 (Rambaut, 2018).

### Gene Content Comparison

Predicted CDS from isolate CPXV-No-H2 were extracted, translated into amino acid sequences, and compared to the CPXV_Br, ECTV_Mos, or VACV_Cop proteins using BLASTp (ncbi-blast+ v2.11.0) (Camacho et al., 2009). To find the closest annotated proteins for all predicted CPXV-No-H2 CDS, every translated CPXV-No-H2 CDS was analyzed by BLASTp search against proteins of the *Poxviridae* family. A BLASTn identity analysis was performed on predicted CPXV-No-H2 CDS that encode proteins with a higher identity to other OPXV proteins than CPXV proteins. When the first hit in BLASTp or BLAStn was CPXV-No-H2 protein or genome, the second hit was used.

### Investigation of Potential Recombination Events

The genome sequence of CPXV-No-H2 was examined for potential recombination events using recombination detection program 4 (RPD4) (Martin et al., 2015) and SimPlot v3.5.1 (Lole et al., 1999). A putative recombinant event was taken into account if it was identified by RDP4 and/or Simplot analysis and the sequence was most similar to the possible minor parental. The whole genome of CPXV-No-H2 was aligned to other OPXV genomes used as putative parentals (AKPV, CPXV_Gri, CPXV_GerMygEK938_17, ECTV_Mos, MPXV_Zaire, and VACV_LC16m8), with MAFFT v1.4.0 (Katoh and Standley, 2013) implemented in Geneious Prime 2020.2.4. Gaps were not removed from the multiple alignments. Similarity plots were performed on the multiple alignments using the SimPlot program (Lole et al., 1999) with default settings. Putative recombination breakpoints were determined by maximization of χ^2^ analysis (Lole et al., 1999; Lim et al., 2011). For recombination analysis with RPD4, seven methods [RDP (Martin and Rybicki, 2000), GENECONV (Padidam et al., 1999), Bootscan (Martin et al., 2005), MaxChi (Smith, 1992), Chimaera (Posada and Crandall, 2001), SiScan (Gibbs et al., 2000), and 3Seq (Boni et al., 2007)] were used to detect potential recombination events. RDP4 was used with the default parameters, except for the option “require topological evidence.” The recombination events that were identified by 6 of 7 methods with significant *p*-values (*p* ≤ 0.01) were considered potential recombinant events. The beginning and end of the breakpoints of these events suggested by RPD4 were used to identify the potential recombinant sequence. When the breakpoints were not identified by RDP4, the range of positions of the breakpoints obtained by Simplot analysis was used. Those potential recombinant sequences were utilized to build an ML tree using RAxML v.8.2.12 (Stamatakis, 2014). Phylogenetic tree incongruence was further used to map potential recombination sequences. Furthermore, a BLASTn identity analysis was performed on those potential recombinant sequences.

## Results

### Genome Assembly and Genome Annotation

**FIGURE 1:**
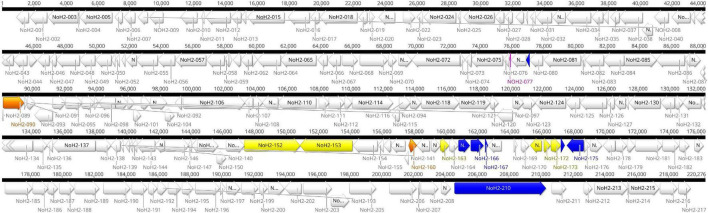
Genome map of CPXV-No-H2. Localization of 217 predicted coding sequences (CDS) and nine putative recombination events in the CPXV-No-H2 genome. Green blocks represent the putative recombination events. Other colors were used to visualize the amino acid sequence similarity between translated CDS to other OPXV proteins: blue blocks represent CDS with a higher similarity to *Alaskapox virus* proteins, yellow blocks represent CDS with a higher similarity to *Ectromelia virus* (ECTV) proteins, orange blocks represent CDS with a higher similarity to *Vaccinia virus* (VACV) proteins, and fuchsia block represents the CDS with a higher similarity to ECTV, VACV, and *Horsepox virus* proteins.

Two large contigs (>1000 bp) were obtained with the hybrid assembly and after removing the host contamination. The average coverage of the major and minor contig was 1502X and 735X, respectively. The mean genomic coverage of CPXV-No-H2 was 1370X. The assembled whole-genome length of CPXV-No-H2 was 220,276 bp. The ITR regions were approximately 7 kbp, and the central region was 206,204 bp. The A+T content of the CPXV-No-H2 genome was 66.6%. Genome annotation predicted 217 potential genes in the CPXV-No-H2 genome (Figure 1 and Supplementary Table 2). The overlapping genes were excluded from the annotation process. However, there were 20 predicted overlapping genes (Supplementary Table 3). Some of them were homologs of CPXV_Br genes (*CPXV004*, *CXPV47*, *CPXV51A*, *CPXV058*, *CPXV078A*, *CPXV096*, *CPXV116*, *CPXV119A*, *CPXV130*, *CPXV152A*, *CPXV160*, *CPXV170*, and *CPXV214*). The whole genome sequence is deposited in GenBank with accession number OM460002.

### Phylogenetic Analysis

**FIGURE 2:**
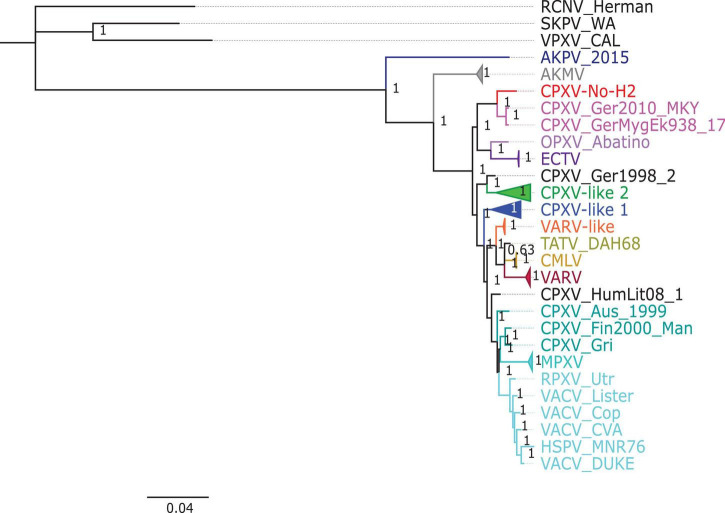
Bayesian inference phylogenetic tree based on 76 orthopoxvirus whole genomes. Posterior probabilities are shown on the right side of each node, and only posterior probabilities above 0.6 are shown. The cowpox virus strains were grouped into different clades: CPXV-like 1, CPXV-like 2, and VARV-like (Franke et al., 2017). The scale bar represents the expected substitutions per site.

**FIGURE 3:**
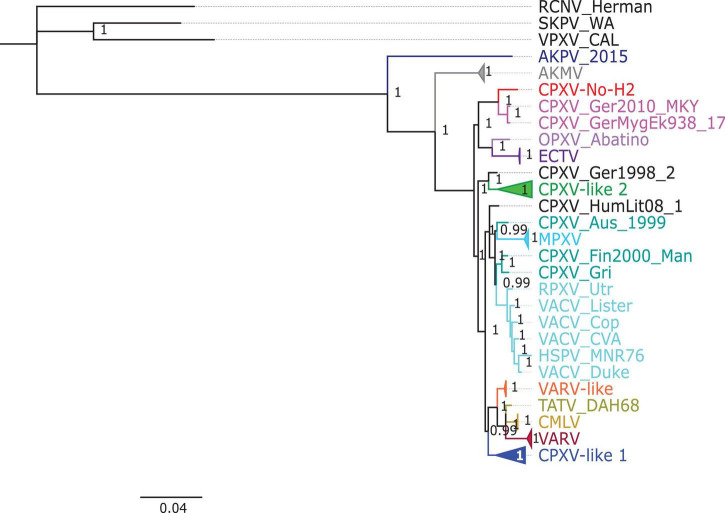
Bayesian inference phylogenetic tree based on 76 orthopoxvirus core genomes. Posterior probabilities are shown on the right side of each node, and only posterior probabilities above 0.6 are shown. The cowpox virus (CPXV) strains were grouped into different clades: CPXV-like 1, CPXV-like 2, and VARV-like (Franke et al., 2017). The scale bar represents the expected substitutions per site.

**FIGURE 4:**
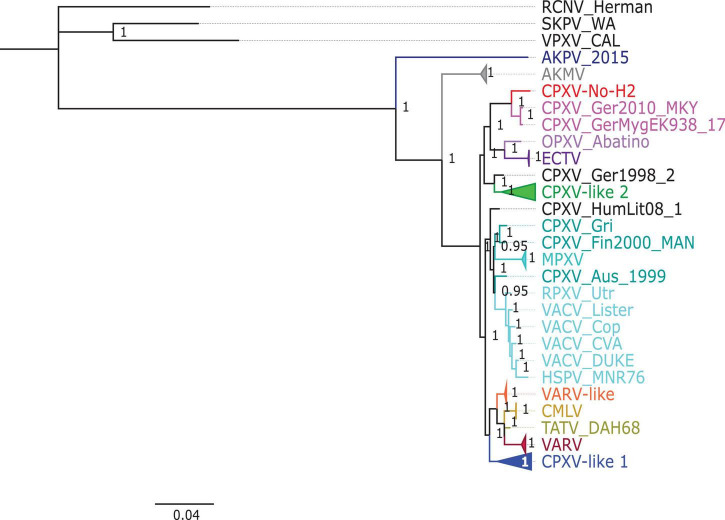
Bayesian inference phylogenetic tree based on 134 orthopoxvirus orthologous genes. Posterior probabilities are shown on the right side of each node, and only posterior probabilities above 0.6 are shown. The cowpox virus (CPXV) strains were grouped into different clades: CPXV-like 1, CPXV-like 2, and VARV-like (Franke et al., 2017). The scale bar represents the expected substitutions per site.

The phylogenetic analysis showed that the ML tree topologies were similar to the phylogenetic trees generated by the BI method, regardless of the alignments used. The BI phylogenetic trees had strong posterior probabilities in most nodes (≥0.95) (Figures 2–4). Unlike the BI trees, the ML trees had low clade support (<70%) in some of the nodes (Supplementary Figures 2–4). The BI phylogenetic trees of 76 OPXV whole genomes, 76 OPXV core genomes, and 134 OPXV orthologous genes are shown in Figures 2–4, respectively. The Old World and New World OPXV were separated into two groups in the phylogenetic trees generated from 76 OPXV whole genomes (Figure 2), 76 OPXV core genomes (Figure 3), and 134 OPXV orthologous genes (Figure 4). Within the Old World OPXV, the strains from the same OPXV species were grouped into clusters, except for CPXV strains that formed more than one cluster. CPXV was divided into clusters: CPXV-like 1, CPXV-like 2, VARV-like, VACV-like, and new clade (Franke et al., 2017). Although the strains of VACV-like did not form a proper cluster, they were closely related VACV (Figures 2–4).

The new clade comprised CPXV-No-H2 and two German CPXV isolates: CPXV_GerMygEK938_17 and CPXV_Ger2010_MKY (posterior probabilities of 1.0 and bootstrap values of 100%) (Figures 2–4 and Supplementary Figures 2–4). The CPXV-No-H2 genome was most similar to the CPXV_GerMygEK_938_17 genome (96.38% identical), and the second most similar virus was CPXV_Ger2010_MKY (96.26% identical), based on the alignment of 76 OPXV whole genomes. The new clade was closely related to the ECTV/Abatino clade. Both clades formed a major clade together (posterior probabilities of 1.0 and bootstrap values > 89%) (Figures 2–4 and Supplementary Figures 2–4). In this study, the new clade (CPXV-No-H2/CPXV_GerMygEK938_17/CPXV_Ger2010_MKY) was tentatively named “ECTV-Abatino-like.”

In phylogenetic trees derived from the 134 OPXV orthologous genes, the ECTV-Abatino-like/ECTV/OPXV Abatino clade clustered with CPXV_Ger1998/CPXV-like 2 clade with a strong posterior probability (1.0), but with a low bootstrap support value (46%) (Figure 4 and Supplementary Figure 4), whereas the phylogeny of the 76 OPXV whole and core genomes showed that the ECTV-Abatino-like/ECTV/OPXV Abatino clade was separated from the other Old World OPXV, which formed a major polyphyletic clade (posterior probability of 1.0 and bootstrap values > 81%) (Figures 2, 3 and Supplementary Figures 2, 3). This major polyphyletic clade was further resolved in two groups: the CPXV_Ger1998/CPXV-like 2 clade (posterior probability of 1.0 and bootstrap values of 100%) and a larger group containing CPXV-like 1, VARV-like, VARV-TATV-CMLV, CPXV_HumLit08, VACV-like, MPXV, RPXV, and VACV clades (posterior probabilities of 1.0 and bootstrap values of 100%) (Figures 2, 3 and Supplementary Figures 2, 3). The clustering within this monophyletic group apparently differs between the tree based on 76 OPXV whole genomes and the trees built from 76 OPXV core genomes and 134 OPXV orthologous genes (Figures 2–4 and Supplementary Figures 2–4). In the former, CPXV-like 1 branches separated from other members of the large polyphyletic group (Figure 2 and Supplementary Figure 2). These members formed a cluster and were further split into two clusters: the VARV-like/TATV/CMLV/VARV cluster and the CPXV_HumLit08/VACV-like/MPXV/RPXV/VACV cluster. Both clusters were supported by strong posterior probabilities (1.0) and bootstrap values (100%) (Figure 2 and Supplementary Figure 2), while 76 OPXV core genomes and 134 OPXV orthologous gene phylogenies grouped CPXV-like 1 into the same cluster with VARV-like/TATV/CMLV/VARV, with posterior probabilities of 0.99 and 1.0 and bootstrap values of 51 and 99%, respectively (Figures 3, 4 and Supplementary Figures 3, 4). Additionally, CPXV_HumLit08, VACV-like, MPXV, RPXV, and VACV were grouped into the same cluster, with posterior probabilities of 1.0 and bootstrap values of 100%.

However irrespective of the aforementioned differences between the whole genome tree on one hand and the core genome and the concatenated 134 orthologous genes on the other, the following topologies were consistent in all the trees generated from the three distinct datasets: (i) ECTV-Abatino-like CPXV clustered closely with ECTV-OPXV Abatino clade, (ii) VACV-like CPXV grouped together with VACV, (iii) VARV-like CPXV clustered closely with VARV-TATV-CMLV clade, (iv) CPXV-like 1 clade is sister to VACV-like clade, and (vi) AKPV/AKMV are intermediate between Old World and New World OPXV.

### Gene Content Comparison

The gene content and organization of the CPXV-No-H2 genome were similar to that of the CPXV_Br and ECTV_Mos genomes. All CPXV_Br genes (excluding ITR genes) were found in the CPXV-No-H2 genome, except for *CPXV221* (encodes CrmD protein) and *CPXV192* (encodes CPXV192 protein). The last gene is truncated in CPXV-No-H2 and overlapped to a major predicted gene. Similarly, comparing CPXV-No-H2 and ECTV_Mos, it was shown that *EVM003/170* (homolog to *CPXV221*) was missing in the CPXV-No-H2 genome. Additionally, the *EVM006* gene (encodes C-type lectin) was absent in the CPXV-No-H2 genome.

The predicted gene *NoH2-154* encodes an intact p4c protein compared to CPXV_Br, whose *p4c* gene is disrupted in two fragments (*CPXV159* and *CPXV161*). Similarly, this gene is fragmented in ECTV_Mos (Chen et al., 2003). The BLASTp analysis for NoH2-154 revealed that the best hit was an inclusion protein III from *Buffalopox virus*, with 87.7% identity. This protein (501 aa) was smaller than the p4c protein from CPXV-No-H2 (512 aa). The next best BLASTp hits were longer proteins of 527 aa from CPXV_Ger2010_MKY and 523 aa from CPXV_GerMygEK938_17, which shared 88.6 and 89.12% identity with p4c protein from CPXV-No-H2, respectively. BLASTn showed that the CPXV-No-H2 *p4c* gene was most similar to the *p4c* gene from CPXV_GerMygEK938_17.

Within ITRs of CPXV-No-H2, five of eight duplicate CPXV_Br genes were found (*CPXV003/227*, *CPXV005/226*, *CPXV006/225*, *CPXV007/224*, and *CPXV008/223*). The terminal *CPXV004* gene was also found in both ITRs of CPXV-No-H2 (*NoH2-A* and *NoH2-T*) (Supplementary Table 3), but they overlapped two major predicted genes (*NoH2-002* and *NoH2-216*). Interestingly, *NoH2-006*, the ortholog of *CPXV009/222*, was found as a single copy downstream of the left ITR of the CPXV-No-H2 genome (Figure 1).

All predicted genes in CPXV-No-H2 were found to have homologs in either CPXV_Br, ECTV_Mos, or VACV_Cop, except for *NoH2*-008 and *NoH2-212*. The translated *NoH2-008* CDS shared 100% amino acid identity with the hypothetical protein CPXV0285 of CPXV_FM2292 (CRL86746.1) and CPXV_Ger2007_Vole (SBN49117.1). The predicted gene *NoH2-212* was a homolog of *CPXV-GRI-K3R* (encodes CrmE protein). The BLASTp analysis of this translated CDS showed that it shared the highest amino acid identity (95.2%) with a CPXV_GerMygEK938_17 protein (hypothetical protein pCPXV003 CAB5514210.1). The *NoH2-212* gene was located upstream of the right ITR of CPXV-No-H2 (Figure 1).

Of the 217 predicted genes of CPXV-No-H2, 17 coded for proteins that were most similar to other OPXV proteins than CPXV proteins. Seven of them shared high similarity to North American OPXV proteins, AKPV, and 10 genes were most similar to Old World OPXV proteins, including ECTV and VACV (Supplementary Table 4). The seven predicted CPXV-No-H2 proteins were most similar (i.e., >92% amino acid identity) to AKPV proteins, including NoH2-079, NoH2-165, NoH2-166, NoH2-167, NoH2-174, NoH2-175, and NoH2-210. The BLASTn analysis of their seven predicted CPXV-No-H2 genes revealed that *NoH2-079*, *NoH2-165*, *NoH2-166*, *NoH2-167*, *NoH2-174*, and *NoH2-210* shared the highest similarity (i.e., > 97% nucleotide identity) with *AKPV-076*, *AKPV-162*, *AKPV-163*, *AKPV-164*, *AKPV-171*, and *AKPV-203*, respectively, whereas *NoH2-175* shared the highest nucleotide similarity with CPXV_GerMygEK938_17/CPXV_Ger2010_MKY. However, the BLASTn analysis of *NoH2-175* with the intergenic region between *NoH2-174* and *NoH2-175* revealed the highest similarity with AKPV (93.98% nucleotide identity).

The six predicted proteins most identical (i.e., > 94% amino acid identity) to ECTV proteins were NoH2-152, NoH2-153, NoH2-163, NoH2-171, NoH2-172, and NoH2-173. At the nucleotide level, *NoH2-152*, *NoH2-153*, *NoH2-171*, *NoH2-172*, and *NoH2-173* had > 95% identity with the corresponding ECTV *EVM127*, *EVM128*, *EVM140*, *EVM141*, and *EVM142* genes. The *NoH2-163* gene, however, was most similar to *CPXV169* from CPXV_GerMygEK938_17 and CPXV_Ger2010_MKY (98.6% identity), whereas the next best BLASTn hit was an ECTV gene with 98.4% identity. The difference between their percent identities was due to one identical nucleotide (Supplementary Figure 1).

The three CPXV-No-H2 predicted proteins most similar to VACV proteins included NoH2-090, NoH2-159, and NoH2-160. The BLASTn search of these predicted genes revealed that *NoH2-159* shared 100% nucleotide identity with VACV, BPXV, and CPXV genomes, and *NoH2-160* was 98.4% identical to VACV LC16m8 (*m8197R*) and VACV LC16mO genes (*mO197R*). The predicted protein of the gene *NoH2-077* was 100% identical to ECTV, HSPV, and VACV proteins. However, the BLASTn of this predicted gene showed that it was 100% identical to CPXV_GerMygEK938_17 genome, but this region was not annotated.

Overlapping genes were excluded from the annotation process. There were 20 overlapping predicted genes (Supplementary Table 3). Fourteen of them were homologs of CPXV_Br (*CPXV004*, *CXPV47*, *CPXV51A*, *CPXV058, CPXV078A*, *CPXV096*, *CPXV116*, *CPXV119A*, *CPXV130*, *CPXV152A*, *CPXV160*, *CPXV170*, and *CPXV214*). Another six overlapping genes did not correspond to any annotated CPXV gene. The BLASTp analysis of the protein encoded by the six overlapping genes revealed that five shared the highest similarity (> 83% amino acid identity) to CMLV_0408151v (NoH2-B), OPXV Abatino (NoH2-H), VACV_CEyV1 (NoH2-G and NoH2-N), or VACV_Lister (NoH2-R) proteins (Supplementary Table 3).

### Recombination Analysis

Previous studies by our group had identified a putative crossover event downstream of the *atip* gene (Okeke et al., 2012). Consequently, the complete CPXV-No-H2 genome was examined for recombination because it contained genomic regions with predicted genes similar to AKPV, ECTV, or VACV genomes. Nine putative recombination events were predicted by RDP4 and Simplot analysis for the CPXV-No-H2 genome (Table 1 and Figure 1). Six potential recombination regions were a result of recombination events between the parentals of AKPV and CPXV (putative recombination events 1–6), two originated from recombination events between the parental ECTV and CPXV (putative recombination events 7 and 8), and one was a product of a recombination event between the parental VACV and CPXV (putative recombination event 9) (Table 1). Within the nine putative recombinant regions in CPXV-No-H2, only one recombinant region (putative recombination event 6) was close to terminal regions, whereas the other eight recombinant regions were located in the central region of the genome.

**TABLE 1:** Predicted recombination events in the CPXV-No-H2 genome using recombination detection program 4 (RPD4) and Simplot analysis.

					RDP4			Simplot	
					
Putative parental strains	Major parental	Minor parental	Recombinant virus	Recombination event	Breakpoint in CPXV-No-H2		Recombination detection programs	Breakpoint interval in CPXV-No-H2	
					Begin (bp)	End (bp)		Begin (bp)	End (bp)
AKPV, CPXV_GerMygEK938_17, CPXV_Gri, CPXV-No-H2	CPXV_GerMygEK938_17	AKPV	CPXV-No-H2	1	76,946	77,244*	RDP, GENECONV, Bootscan, MaxChi, Chimaera, 3Seq	76,679–76,957	77,201–77,208
	CPXV_GerMygEK938_17	AKPV	CPXV-No-H2	2	77,741	78,243	RDP, GENECONV, Bootscan, MaxChi, Chimaera, 3Seq	77,717–77,765	78,237–78,399
	CPXV_GerMygEK938_17	AKPV	CPXV-No-H2	3	150,156	154,530	GENECONV, Bootscan, MaxChi, Chimaera, SiScan, 3Seq	150041–150158	154,524–154,570
	CPXV_GerMygEK938_17	AKPV	CPXV-No-H2	4	160,988*	162,917*	RDP, GENECONV, Bootscan, MaxChi, Chimaera, SiScan, 3Seq	160,774–160,878	162,909–162,948
	CPXV_GerMygEK938_17	AKPV	CPXV-No-H2	5	165,874	168,063	RDP, GENECONV, Bootscan, MaxChi, Chimaera, SiScan, 3Seq	165,828–165,878	168,042–168,066
	CPXV_GerMygEK938_17	AKPV	CPXV-No-H2	6	-	-	-	204,960–204,977	209,498–209,901
ECTV_Mos, CPXV_GerMygEK938_17, CPXV_Gri, CPXV-No-H2	CPXV_GerMygEK938_17	ECTV_Mos	CPXV-No-H2	7	150,119	153,968	GENECONV, Bootscan, MaxChi, Chimaera, SiScan, 3Seq	149,993–150,158	153,952–154,180
	CPXV_GerMygEK938_17	ECTV_Mos	CPXV-No-H2	8	165,847	167,892	RDP, GENECONV, Bootscan, MaxChi, Chimaera, SiScan, 3Seq	165,678–165,855	167,879–167,943
VACV_LC16m8, CPXV_GerMygEK938_17, CPXV_Gri, CPXV-No-H2	CPXV_GerMygEK938_17	VACV_LC16m8	CPXV-No-H2	9	164,419*	165,036*	RDP, Bootscan, MaxChi, Chimaera, SiScan, 3Seq	164,399–164,525	164,756–164,768

*The breakpoint that was undetermined is marked with an asterisk. AKPV, Alaskapox virus; CPXV, Cowpox virus; ECTV, Ectromelia virus; VACV, Vaccinia virus. The breakpoint that was undetermined is marked with an asterisk.*

The first potential recombinant region in the CPXV-No-H2 genome (putative recombination event 1) comprised the *NoH2-079* gene and started from position 76,946 bp in the CPXV-No-H2. The ending breaking could be between positions 77,201 and 77,208 bp in the CPXV-No-H2 genome based on Simplot analysis. The next potential recombinant region (putative recombination event 2) was almost 500 bp downstream of the first one. It was located between 77,741 and 78,243 bp in the CPXV-No-H2 genome and contained parts of *NoH2-080* and *NoH2-081*. These two putative recombinant regions shared the highest similarity to the AKPV genome (>98% identical) (Supplementary Table 5).

The third potential recombinant region (putative recombination event 3) spanned approximately 4,500 bp, from position 150,156 to 154,530 bp in the CPXV-No-H2 genome. However, it overlapped with the predicted recombinant region between the parental ECTV and CPXV (putative recombination event 7), located between 150,119 and 153,968 bp in the CPXV-No-H2 genome. The latter encompassed only two genes, *NoH2-152* and *No-153*, compared to the former that also contained part of the *NoH2-154* gene. The BLASTn analysis of the third potential recombinant region revealed the highest similarity with the AKPV genome (96.89% identical), whereas the putative recombinant region between the parental ECTV and CPXV was most similar to the ECTV genomes, with 97.93% nucleotide identity (Supplementary Table 5).

The fourth potential recombinant region in the CPXV-No-H2 genome (putative recombination event 4) included the genes *NoH2-165*, *NoH2-166*, and *NoH2-167* and part of *NoH2-168*. The Simplot analysis revealed that the beginning and ending breakpoints were located between 160,774 and 160,878 bp and between 162,909 and 162,948 bp in the CPXV-No-H2 genome, respectively. This genomic region was most similar to the AKPV genome, sharing 97.66% nucleotide identity (Supplementary Table 5). The fifth potential recombinant region (putative recombination event 5) started from 165,874 to 168,063 bp in the CPXV-No-H2 genome. It overlapped with another putative recombinant region between the parental ECTV and CPXV (putative recombination event 8), which was located between 165,847 and 167,892 bp in the CPXV-No-H2 genome. Both regions contained part of *NoH2-171*, *NoH2-172*, *NoH2-173*, and *NoH2-174* and part of *NoH2-175*. The BLASTn analysis of these two putative recombination regions revealed that the first hit was the AKPV genome, with > 97% nucleotide identity (Supplementary Table 5).

A sixth potential recombinant event between the parental AKPV and CPXV (putative recombination event 6) was detected only by Simplot analysis. The cross-over points lay between 204,960 and 204,977 bp and between 209,488 and 209,901 bp in the CPXV-No-H2 genome. It contained a major part of the *NoH2-210* gene, which was most similar to AKPV-203 (97.15% identical) and also shared similarity with the Murmansk-007 gene (91.44% identical). Furthermore, this putative recombinant sequence showed its highest identity with the AKPV genome (98.4% identical), followed by the *Murmansk microtuspox virus* genome (91.44% identical).

One putative recombination event between the parental VACV and CPXV (putative recombination event 9) was detected. The breakpoints were undetermined by RDP4, but the Simplot analysis revealed that the putative recombinant sequence started from position 164,400–164,525 bp and ended at position 164,756–164,768 bp in the CPXV-No-H2 genome. This region contained a small part of *NoH2-169* and a major part of *NoH2-170*. The latter gene shared 94.44% identity with the genes of CPXV and RPXV and the VACV strains, such as Lister, Cantagalo, CVA, and NYCBH.

The phylogenetic analysis of the six putative recombinant regions between the parental AKPV and CPXV showed that CPXV-No-H2 clustered with AKPV with a bootstrap support of > 91%, except for the phylogenetic tree based on the fifth putative recombinant region (165,874–168,063 bp in the CPXV-No-H2 genome), where CPXV-No-H2 clustered with ECTV with a low bootstrap support (55%), and they were grouped with AKPV (bootstrap value of 100%) (Supplementary Figures 5–10). CPXV-No-H2 likewise clustered with ECTV in the phylogenetic tree generated from the potential recombinant region between the parental ECTV and CPXV (165,847–167,892 bp in the CPXV-No-H2 genome) that overlapped the fifth putative recombinant region (Supplementary Figure 12). Unlike the previous phylogenetic tree, the bootstrap support for this clade was higher (93%) though.

Based on the phylogenetic analysis of the putative recombinant sequence between the parental ECTV and CPXV (150,119–153,968 bp in the CPXV-No-H2 genome), CPXV-No-H2 formed a cluster with ECTV (Supplementary Figure 11). This cluster was most closely related to AKPV and formed a major clade, with AKPV and AKMV, separating them from other Old World OPXV. However, the phylogenetic tree of the recombinant region between the parental AKPV and CPXV (150,156–154,530 bp in the CPXV-No-H2 genome), which overlapped that recombinant region, clustered CPXV-No-H2 with AKPV, and both isolates were closely related to ECTV (Supplementary Figure 7). The phylogenetic tree based on the putative recombinant sequence between the parental VACV and CPXV placed CPXV-No-H2 inside the VACV cluster (Supplementary Figure 13).

## Discussion

CPXV-No-H2 is an isolate from a human in Northern Norway that was classified as an atypical CPXV based on ATI phenotype, sequence of the *atip* and *p4c* genes, and Hind III restriction map (Hansen et al., 2009; Okeke et al., 2012). Our phylogeny analysis indicated that CPXV-No-H2 is most closely related to the German CPXV isolates CPXV_GerMygEK938_17 and CPXV_Ger2010_MKY (Figures 2–4). Similarly, phylogenetic analysis based on the *HA* gene also resolved CPXV_Ger2010_MKY and CPXV-No-H2 in the same cluster (Kalthoff et al., 2014). The three CPXV isolates (CPXV_GerMygEK938_17, CPXV_Ger2010_MKY, and CPXV-No-H2) may be part of a novel CPXV lineage separated from the other CPXV strains. It was previously suggested that CPXV_Ger2010_MKY and CPXV_GerMygEK938_17 were part of a new cluster provisionally called CPXV-like 3 (Franke et al., 2017; Jeske et al., 2019). However, this cluster was supported by a low bootstrap value (Jeske et al., 2019). The phylogenetic analysis reported in our study indicated that the new clade (CPXV-No-H2/CPXV_GerMygEK938_17/CPXV_Ger2010_MKY) was more closely related to ECTV and OPXV Abatino than other OPXVs, with strong posterior probabilities and bootstrap values (Figures 2–4 and Supplementary Figures 2–4). Thus, we tentatively named this clade as “ECTV-Abatino-like.”

The ECTV-Abatino-like/ECTV/OPXV Abatino clade was separated from the Old World OPXV in 76 OPXV whole- and core-genome phylogenetic trees, while a phylogenetic tree based on 134 OPXV orthologous genes showed that this clade clustered closely with CPXV_Ger1998/CPXV-like 2 but with poor bootstrap support (46%). We suggest that the separation of ECTV-Abatino-like/ECTV/OPXV Abatino from the other Old World OPXV may be due to the presence of some genes (or genomic regions) located in the core genome, which are not included within the 134 OPXV orthologous genes. A previous study showed that CPXV_GerMygEK938_17/CPXV_Ger2010_MKY/ECTV/OPXV Abatino clustered with CPXV-like 2 but with low bootstrap support (< 70%) (Jeske et al., 2019), although AKPV was not included in their phylogenetic analysis compared to our study that included AKPV and more OPXV strains. When AKPV was excluded from the construction of our phylogenetic trees, the ECTV-Abatino-like/ECTV/OPXV Abatino clade clustered with CPXV_Ger1998/CPXV-like 2 in 75 OPXV whole- and core-genome phylogenetic trees but with low bootstrap support (Supplementary Figures 14–17). In contrast, the bootstrap value in the node that clustered these clades increased from 46 to 82% in the phylogenetic tree based on 134 OPXV orthologous genes (Supplementary Figures 18, 19). We suspect that the genes or genomic regions that separated those clades have homologs in AKPV—for instance, homologs of *NoH2-166*, *NoH2-167*, *NoH2-174*, and *NoH2-210* genes, which were most similar to the AKPV genes, were not included in the construction of the phylogenetic tree based on 134 OPXV orthologous genes.

In fact, CPXV-No-H2 has a mosaic genome with genes most similar to the OPXV genes from the Old World, including ECTV and VACV, and the North America, AKPV. Previously, we have shown that the *atip* gene from CPXV-No-H2 displayed the highest similarity to the corresponding ECTV gene, and the insertion of the ECTV *atip* gene may be a result of the recombination between CPXV and ECTV or an ECTV-like virus (Okeke et al., 2012). Our present study suggested similar findings and indicated that CPXV-No-H2 has also undergone recombination events between AKPV and VACV. A recombination between OPXVs has been reported by others (Gubser et al., 2004; Coulson and Upton, 2011; Qin et al., 2011, 2015; Okeke et al., 2012; Smithson et al., 2014, 2017a; Franke et al., 2017; Gao et al., 2018; Gigante et al., 2019).

CPXV-No-H2 displays recombination events with OPXVs that were isolated from different places and species. CPXV-No-H2 is a strain from Northern Norway (Okeke et al., 2012). Its closest relatives CPXV_GerMygEK938_17 and CPXV_Ger2010_MKY were isolated in Germany, but they were isolated from different species: bank vole and cotton-top tamarin, respectively (Kalthoff et al., 2014; Jeske et al., 2019). It was suggested that the infection of cotton-top tamarin was mediated by bank vole infected with CPXV (Jeske et al., 2019; Weber et al., 2020). In contrast, AKPV was isolated from a human patient in North America (Alaska, the United States). The patient‘s infection source is unknown, but it is presumable that she was infected by a small mammal (Springer et al., 2017; Gigante et al., 2019). VACV and ECTV have been reported around the world (Dumbell and Richardson, 1993; Miranda et al., 2017; Mavian et al., 2021). ECTV infects laboratory mice worldwide (Trentin and Briody, 1953; Mavian et al., 2021; Wang et al., 2021). The first discovered ECTV strain, ECTV_Hampstead, was isolated in the United Kingdom and was the progenitor of the European outbreaks. Only one ECTV strain (ECTV_MouKre) was isolated from a wild mouse in Germany (Mavian et al., 2021). The worldwide presence of ECTV in animals suggests their presence also in Norwegian fauna and hence the possibility to recombine with CPXV.

Among the nine potential recombination events in the CPXV-No-H2 genome, two potential recombination events with the parental AKPV (putative recombination events 3 and 5) overlap with two potential recombination events with the parental ECTV (putative recombination events 6 and 7). Interestingly, in the same position of these recombinant regions, AKPV has undergone a potential recombination with ECTV, and it was suggested that ECTV contains an AKPV-like sequence (Gigante et al., 2019).

These recombinant regions (putative recombination events 5 and 8) contain the *atip* gene, which is one of the three genes (*atip*, *p4c*, and *A27L*) required for the formation of the V^+^ ATI phenotype (Patel and Pickup, 1987; McKelvey et al., 2002; Howard et al., 2010). CPXV-No-H2 contains an intact ECTV-like *atip*, *p4c*, and *A27L* genes. Those latter genes were most similar to CPXV_GerMygEK938_17 genes. CPXV-No-H2 produces mainly virions encrusted on the surface of ATI (V^+/^) similar to ECTV_Hampstead, which produces both V^+^ and V^+/^ ATI phenotype (Ichihashi and Matsumoto, 1966; Okeke et al., 2012; Mavian et al., 2021). ECTV_Hampstead encodes a full-length p4c protein compared to other ECTV isolates with V^–^ ATI phenotype. Besides this, it contains the *atip* and *A27L* genes (Mavian et al., 2021). AKPV and CPXV_Ger2010_MKY also comprise these three genes and produce the V^+^ ATI phenotype (Franke et al., 2017; Springer et al., 2017; Gigante et al., 2019). There is no report of the production of ATI bodies in CPXV_GerMygEK938_17; however, its *atip*, *p4c*, and *A27L* genes are most similar to those of CPXV_Ger2010_MKY.

The potential recombination event between the parental AKPV and CPXV (putative recombinant event 6) located close to the terminal region contains part of the *NoH2-210* gene that shared similarity with *AKPV-203* and the Murmansk gene. *AKPV-203* is one of the three AKPV genes that may be introduced from/to Murmansk poxvirus by recombination (Gigante et al., 2019). Murmansk is a non-OPXV that belongs to the genus *Centapoxvirus* that was isolated in Murmansk, Russia (Smithson et al., 2017a). In three of the six recombination events with the parental AKPV (putative recombination events 1, 4, and 6), it seems that CPXV-No-H2 contains AKPV-like sequences rather than AKPV containing CPXV-No-H2-like sequences because the phylogenetic trees showed that CPXV-No-H2 is not part of the ECTV-Abatino-like clade and was placed next to AKPV (Supplementary Figures 5, 8, 10). In contrast, the overlapping recombinant regions seem to be CPXV-No-H2-like sequences that were introduced to AKPV based on the phylogenetic tree and the sequence similarity (Supplementary Figures 7, 9, 11, 12).

Reconstructing the evolutionary history of CPXV-No-H2 is difficult since it displays several potential recombination events with different OPXVs, especially when it is suspected that recombination events occurred between these OPXVs (such as AKPV and ECTV) (Gigante et al., 2019). Additionally, these OPXVs were isolated from different continents (Springer et al., 2017; Mavian et al., 2021). One plausible hypothesis about the mosaic genome of CPXV-No-H2 is that the CPXV_GerMygEK938_17-like virus was probably circulating in a population of rodents in Europe, and it underwent recombination with the AKPV-like virus. The resultant virus, CPXV-No-H2-like virus, could have suffered genomic changes and adapted to mice, which could be the possible ancestor of ECTV. The origin of ECTV from the CPXV-like ancestor was previously proposed (Jeske et al., 2019) since ECTV has a shorter genome (ranging from 204 to 208 kbp) and reduced number of genes compared to CPXV that has the largest genome among OPXVs, about 220 kbp (Chen et al., 2003; Hendrickson et al., 2010; Carroll et al., 2011; Dabrowski et al., 2013; Mavian et al., 2014, 2021). Our results suggest that CPXV-No-H2 could be derived from a CPXV_GerMygEK938_17-like virus because (1) CPXV_GerMygEK938_17 shares the highest similarity with CPXV-No-H2, (2) it did not show any significant recombination event (Kalthoff et al., 2014; Jeske et al., 2019), (3) none of the seven recombination regions in CPXV-No-H2 was highly similar to either CPXV_GerMygEK938_17 or CPXV_Ger2010_MKY, (4) there is high similarity between their *p4c* and *A27L* genes, (5) its place of isolation was also in Europe, and (6) we speculated that it has V^+^ ATI phenotype similar to CPXV_Ger2010_MKY due to the similarity between their *atip*, *p4c*, and *A27L* genes.

The recombination may have occurred between CPXV_GerMygEK938_17-like virus and AKPV-like virus rather than ECTV-like virus because, aside from two recombination events with the parental AKPV that overlapped a recombination event with the parental ECTV, there are other four recombinant events with the parental AKPV which cannot be viewed as a simple coincidence. In addition, the two suspected recombination regions in the ECTV genome (Gigante et al., 2019) were more similar to CPXV-No-H2 than AKPV (data not shown). Furthermore, hypothetically, CPXV_GerMygEK938_17 may produce V^+^ ATI similar to AKPV, while CPXV-No-H2 and ECTV_Hampstead produce both V^+^ and V^+/^ ATI phenotypes (Ichihashi and Matsumoto, 1966; Okeke et al., 2012; Mavian et al., 2021). It seems that the putative progeny virus, CPXV-No-H2-like virus, may have reduced its ability to embed virions into ATI bodies. This was also observed in the derivates of ECTV_Hampstead that produces the V^–^ ATI phenotype (Mavian et al., 2021). We speculated that the recombination between CPXV_GerMygEK938_17-like virus and AKPV-like virus could take place in a rodent in Europe because AKPV contains genes from a Russian poxvirus, Murmansk, which was isolated from a root vole (Smithson et al., 2017a; Gigante et al., 2019), and CPXV_GerMygEK938_17 was isolated from bank vole in Europe (Jeske et al., 2019). Furthermore, CPXV-No-H2 was isolated in Europe, likewise with CPXV_Ger2010_MKY and ECTV_Hampstead (the source of the European outbreaks) (Hansen et al., 2009; Okeke et al., 2012; Kalthoff et al., 2014; Mavian et al., 2021).

However, it is pertinent to note that recombination detection programs predict hypothetical recombination events across genomes, and the outputs are sensitive to input parameter settings, particularly the sliding window size. To increase the likelihood of putative recombination events being real, we recommend the following: (i) use of these programs at default settings, (ii) identification of the exact recombination event by at least two different programs and algorithms, (iii) discountenance of recombination events without very high statistical support, (iv) confirmation of recombination breakpoints by manual inspection of similarity plots, and (v) incongruence of phylogenetic trees.

Another explanation for the presence of the OPXV-like genomic regions in CPXV-No-H2 could be symplesiomorphy because most genomic regions were similar to more than one taxon—for instance, the two CPXV-No-H2 genomic regions that were similar to ECTV and AKPV may be inherited from a common ancestral virus, likewise with the AKPV-like genomic region that contains part of the *NoH2-210* similar to AKPV and Murmansk. However, symplesiomorphy does not explain the presence of the AKPV-like genomic region of 2,150 bp in CPXV-No-H2, which did not share high similarity with other taxa. The only plausible explanation is that CPXV-No-H2 may have obtained this sequence from an AKPV-like virus by recombination.

Overall, the genetic analysis of the atypical CPXV-No-H2 suggested that it contains sequences similar to other OPXVs, and one of the plausible explanations for their presence was recombination events with other OPXVs. In addition, CPXV-No-H2 is part of a new CPXV clade that was more phylogenetically related to ECTV and OPXV Abatino than other CPXV strains. Our findings provide some insight into the evolutionary history of CPXV and strongly support the genetic heterogeneity of the species CPXV. The discovery of new CPXV isolates and their phylogenetic relationship with OPXVs as well their genomic characterization will contribute to the further elucidation of the complex evolutionary history of CPXV.

## Data Availability Statement

The original contributions presented in the study are publicly available. This data can be found here: https://www.ncbi.nlm.nih.gov/genbank/, OM460002.

## Author Contributions

DD-C conducted the experiments, analyzed the data, and wrote the manuscript. MO and UM conceptualized the study, supervised the design and execution of the project, and wrote the manuscript. AB and AN contributed to data interpretation and revision of the manuscript for improved intellectual content. All authors contributed to the article and approved the submitted version.

## Conflict of Interest

The authors declare that the research was conducted in the absence of any commercial or financial relationships that could be construed as a potential conflict of interest.

## Publisher’s Note

All claims expressed in this article are solely those of the authors and do not necessarily represent those of their affiliated organizations, or those of the publisher, the editors and the reviewers. Any product that may be evaluated in this article, or claim that may be made by its manufacturer, is not guaranteed or endorsed by the publisher.

## Footnotes

1
https://talk.ictvonline.org/taxonomy/

2
https://blast.ncbi.nlm.nih.gov/Blast.cgi
