# Supplementary material for: Genomic Sequencing and Analysis of a Novel Human Cowpox Virus With Mosaic Sequences From North America and Old World Orthopoxvirus
Source: Front Microbiol. 2022 May 3;13:868887. doi: 10.3389/fmicb.2022.868887 (PMC9112427; doi:10.3389/fmicb.2022.868887)
Supplement: Supplementary file 2 [file Data_Sheet_1.pdf]

Supplementary Material

1 Supplementary Figures

Supplementary Figure S1. Schematic diagram of alignment of CPXV-No-H2, CPXV_GerMygEK938_17, CPXV_Ger2010_MKY and ECTV_Mos showing the *NoH2-163* gene and their homologs, using Geneious software 2021.2.2. Grey regions indicate conserved bases while colors (red, blue, yellow, green) indicate differences (A, C, G, T, respectively) from CPXV-No-H2.

Supplementary Figure S2. Maximum-Likelihood phylogenetic tree based on 76 orthopoxvirus whole genomes. Bootstrap values were determined from 1000 replica sampling. *Cowpox virus* (CPXV) strains were grouped into different clades: CPXV-like 1, CPXV-like 2, and VARV-like (Franke et al., 2017). The scale bar represents expected substitutions per site.

Supplementary Figure S3. Maximum-Likelihood phylogenetic tree on 76 orthopoxvirus core genomes. Bootstrap values were determined from 1000 replica sampling. *Cowpox virus* (CPXV) strains were grouped into different clades: CPXV-like 1, CPXV-like 2, and VARV-like (Franke et al., 2017). The scale bar represents expected substitutions per site.

Supplementary Figure S4. Maximum-Likelihood phylogenetic tree of 134 orthopoxvirus orthologous genes. Bootstrap values were determined from 1000 replica sampling. *Cowpox virus* (CPXV) strains were grouped into different clades: CPXV-like 1, CPXV-like 2, and VARV-like (Franke et al., 2017). The scale bar represents expected substitutions per site.

Supplementary Figure S5. Maximum-Likelihood phylogenetic tree based on the putative recombinant region 1 between the parental AKPV and CPXV (potential recombinant event 1). Bootstrap values were determined from 1000 replica sampling. Clades are identified with colors.

Supplementary Figure S6. Maximum-Likelihood phylogenetic tree based on the putative recombinant region 2 between the parental AKPV and CPXV (potential recombinant event 2). Bootstrap values were determined from 1000 replica sampling. Clades are identified with colors.

Supplementary Figure S7. Maximum-Likelihood phylogenetic tree based on the putative recombinant region 3 between the parental AKPV and CPXV (potential recombinant event 3). Bootstrap values were determined from 1000 replica sampling. Clades are identified with colors.

Supplementary Figure S8. Maximum-Likelihood phylogenetic tree based on the putative recombinant region 4 between the parental AKPV and CPXV (potential recombinant event 4). Bootstrap values were determined from 1000 replica sampling. Clades are identified with colors.

Supplementary Figure S9. Maximum-Likelihood phylogenetic tree based on the putative recombinant region 5 between the parental AKPV and CPXV (potential recombinant event 5). Bootstrap values were determined from 1000 replica sampling. Clades are identified with colors.

Supplementary Figure S10. Maximum-Likelihood phylogenetic tree based on the putative recombinant region 6 between the parental AKPV and CPXV (potential recombinant event 6). Bootstrap values were determined from 1000 replica sampling. Clades are identified with colors.

Supplementary Figure S11. Maximum-Likelihood phylogenetic tree based on the putative recombinant region 1 between the parental ECTV and CPXV (potential recombinant event 7). Bootstrap values were determined from 1000 replica sampling. Clades are identified with colors.

Supplementary Figure S12. Maximum-Likelihood phylogenetic tree based on the putative recombinant region 2 between the parental ECTV and CPXV (potential recombinant event 8). Bootstrap values were determined from 1000 replica sampling. Clades are identified with colors.

Supplementary Figure S13. Maximum-Likelihood phylogenetic tree based on the putative recombinant region between the parental VACV and CPXV (potential recombinant event 9). Bootstrap values were determined from 1000 replica sampling. Clades are identified with colors.

Supplementary Figure S14. Maximum-Likelihood phylogenetic tree based on 75 OPXV whole genomes. Bootstrap values were determined from 1000 replica sampling. Clades are identified with colors.

Supplementary Figure S15. Bayesian Inference phylogenetic tree based on 75 OPXV whole genomes. Clades are identified with colors.

Supplementary Figure S16. Maximum-Likelihood phylogenetic tree based on 75 OPXV core genomes. Bootstrap values were determined from 1000 replica sampling. Clades are identified with colors.

Supplementary Figure S17. Bayesian Inference phylogenetic tree based on 75 OPXV core genomes. Clades are identified with colors.

Supplementary Figure S18. Maximum-Likelihood phylogenetic tree based on 134 orthologous genes from 75 OPXV genomes. Bootstrap values were determined from 1000 replica sampling. Clades are identified with colors.

Supplementary Figure S19. Bayesian Inference phylogenetic tree based on 134 orthologous genes from 75 OPXV genomes. Clades are identified with colors.

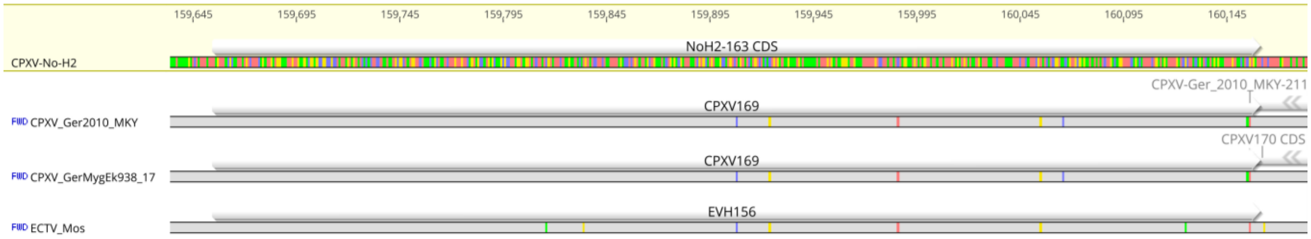

Supplementary Figure S1. Schematic diagram of alignment of CPXV-No-H2, CPXV_GerMygEK938_17, CPXV_Ger2010_MKY and ECTV_Mos showing the *NoH2-163* gene and their homologs, using Geneious software 2021.2.2. Grey regions indicate conserved bases while colors (red, blue, yellow, green) indicate differences (A, C, G, T, respectively) from CPXV-No-H2.

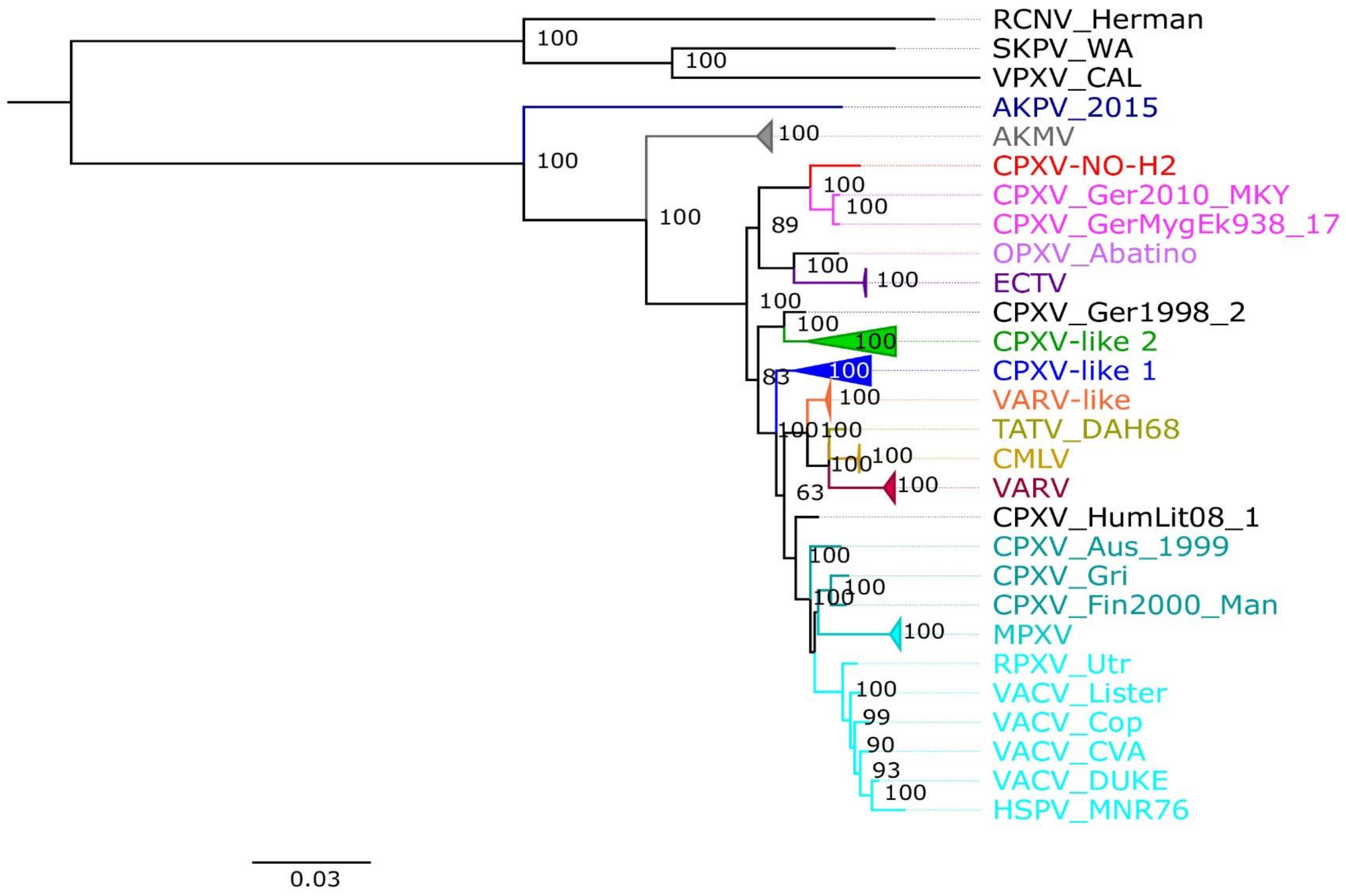

Supplementary Figure S2. Maximum-Likelihood phylogenetic tree based on 76 orthopoxvirus whole genomes. Bootstrap values were determined from 1000 replica sampling. *Cowpox virus* (CPXV) strains were grouped into different clades: CPXV-like 1, CPXV-like 2, and VARV-like (Franke et al., 2017). The scale bar represents expected substitutions per site.

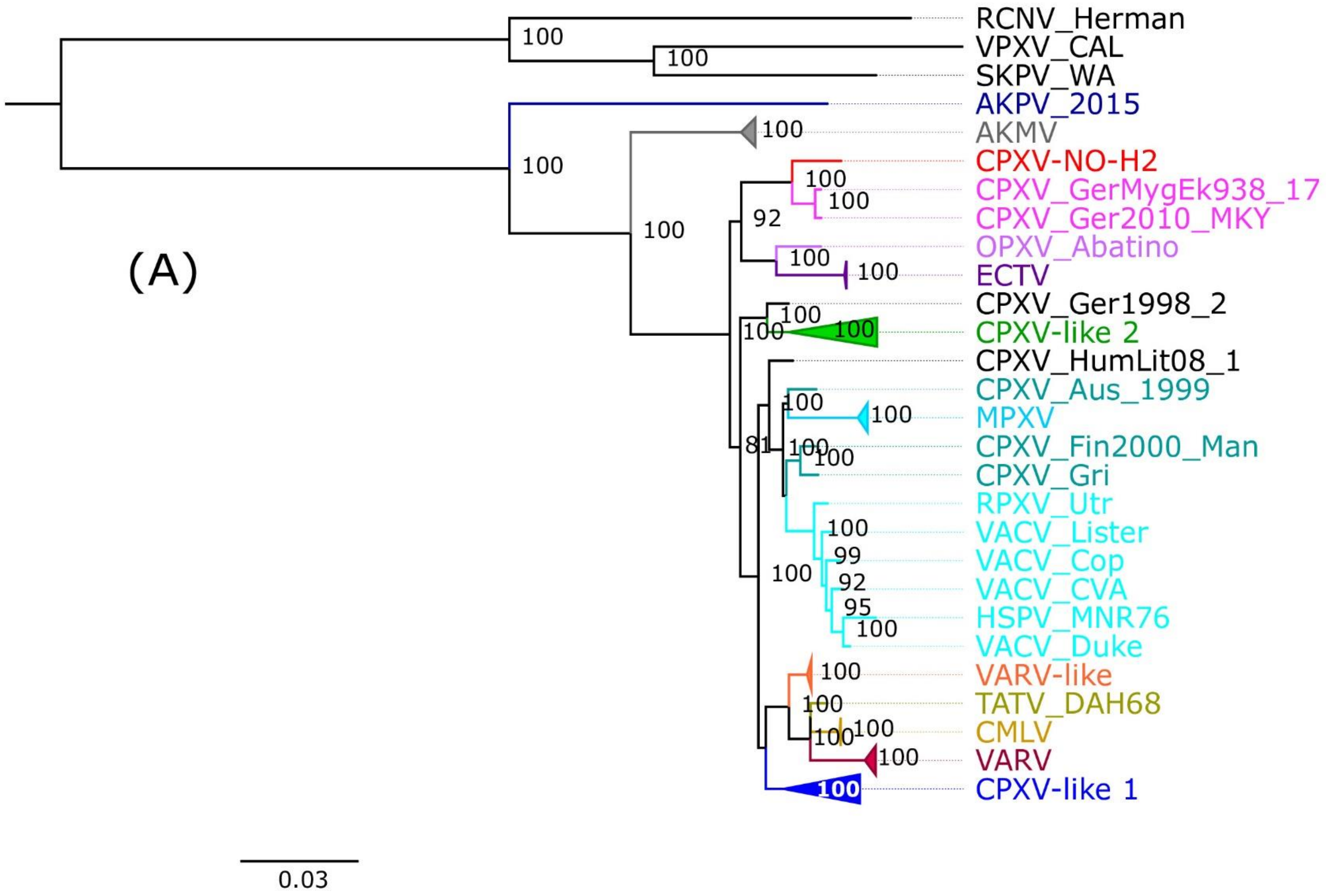

Supplementary Figure S3. Maximum-Likelihood phylogenetic tree on 76 orthopoxvirus core genomes. Bootstrap values were determined from 1000 replica sampling. *Cowpox virus* (CPXV) strains were grouped into different clades: CPXV-like 1, CPXV-like 2, and VARV-like (Franke et al., 2017). The scale bar represents expected substitutions per site.

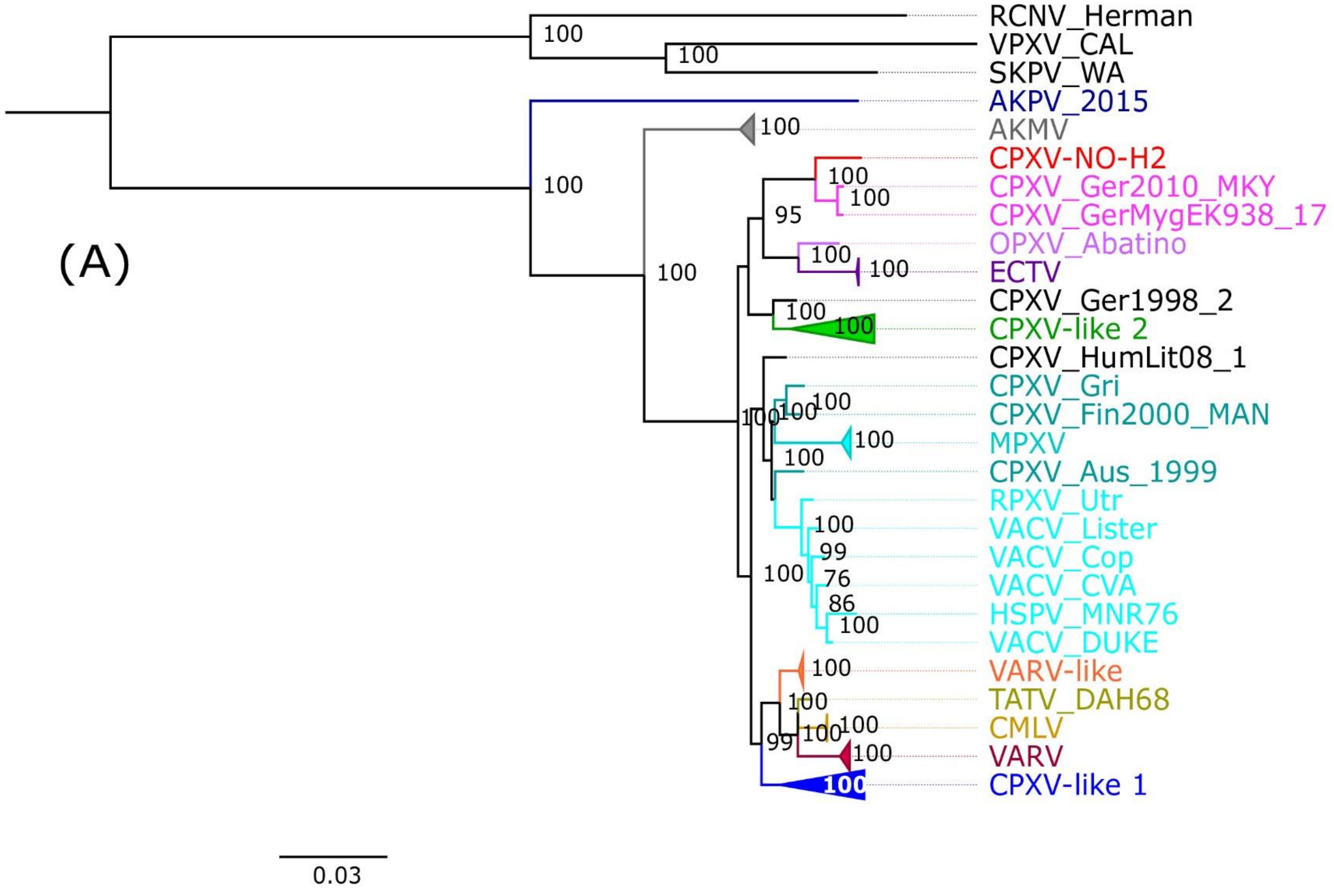

Supplementary Figure S4. Maximum-Likelihood phylogenetic tree of 134 orthopoxvirus orthologous genes. Bootstrap values were determined from 1000 replica sampling. *Cowpox virus* (CPXV) strains were grouped into different clades: CPXV-like 1, CPXV-like 2, and VARV-like (Franke et al., 2017). The scale bar represents expected substitutions per site.

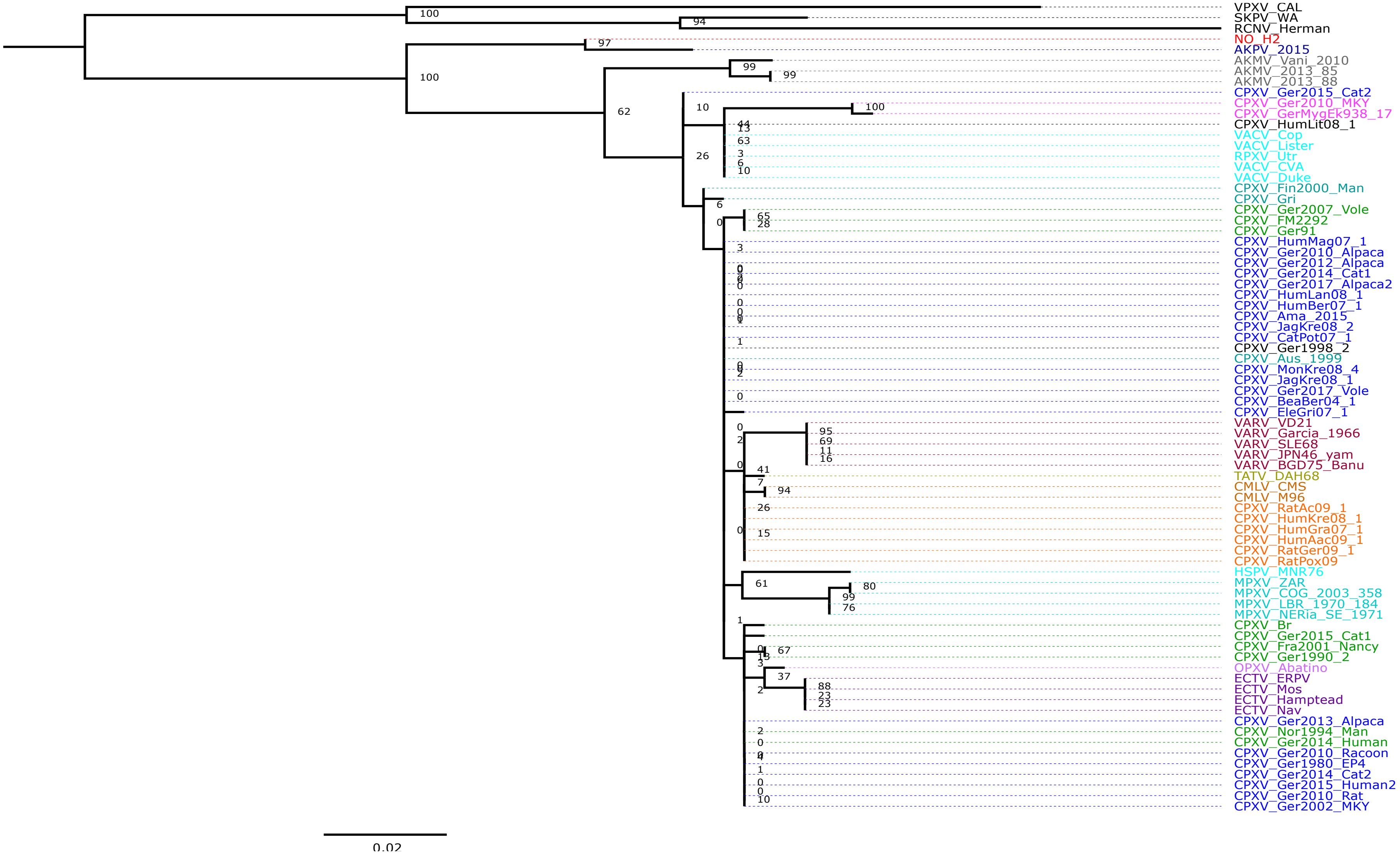

Supplementary Figure S5. Maximum-Likelihood phylogenetic tree based on the putative recombinant region 1 between the parental AKPV and CPXV (potential recombinant event 1). Bootstrap values were determined from 1000 replica sampling. Clades are identified with colors.

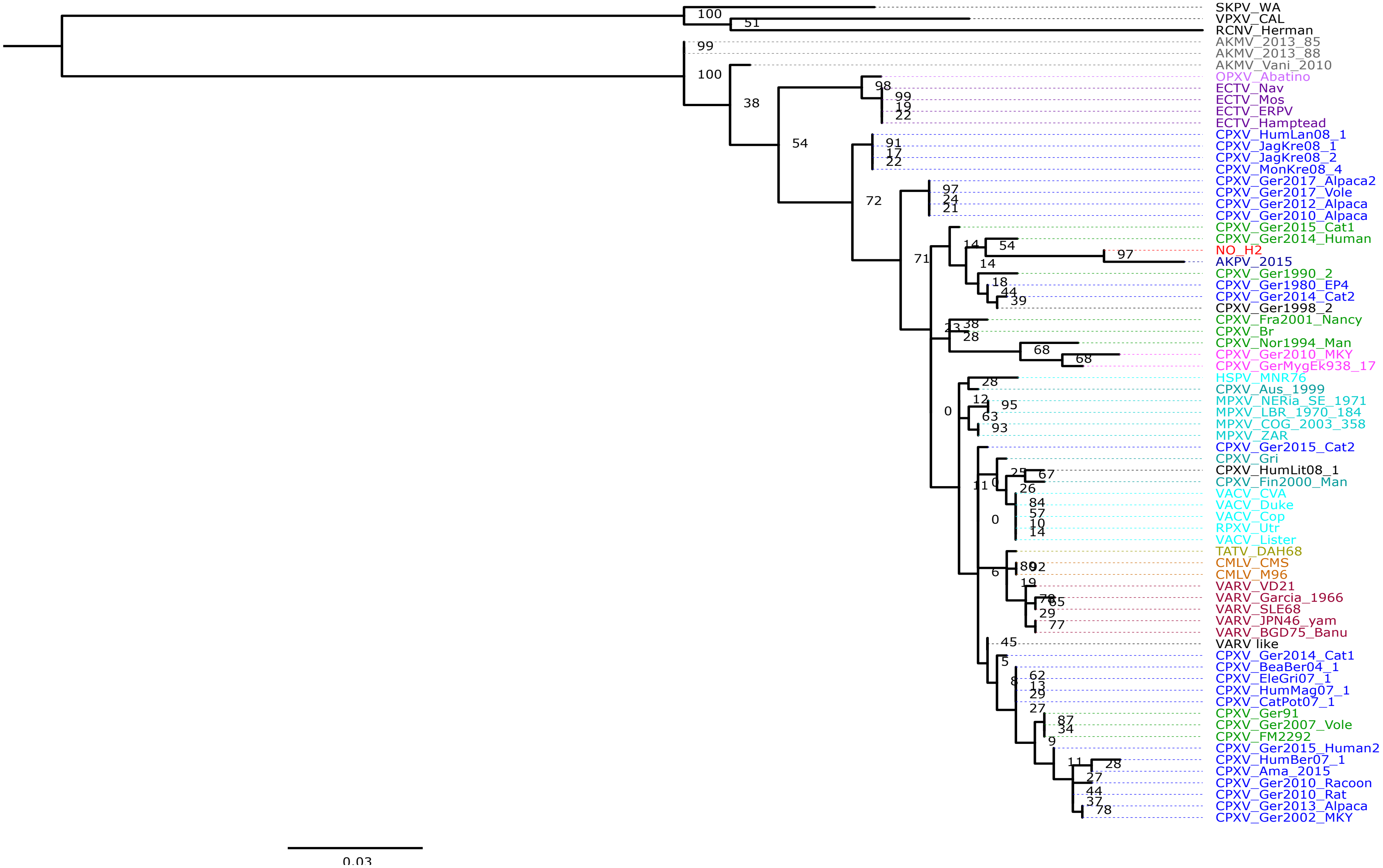

Supplementary Figure S6. Maximum-Likelihood phylogenetic tree based on the putative recombinant region 2 between the parental AKPV and CPXV (potential recombinant event 2). Bootstrap values were determined from 1000 replica sampling. Clades are identified with colors.

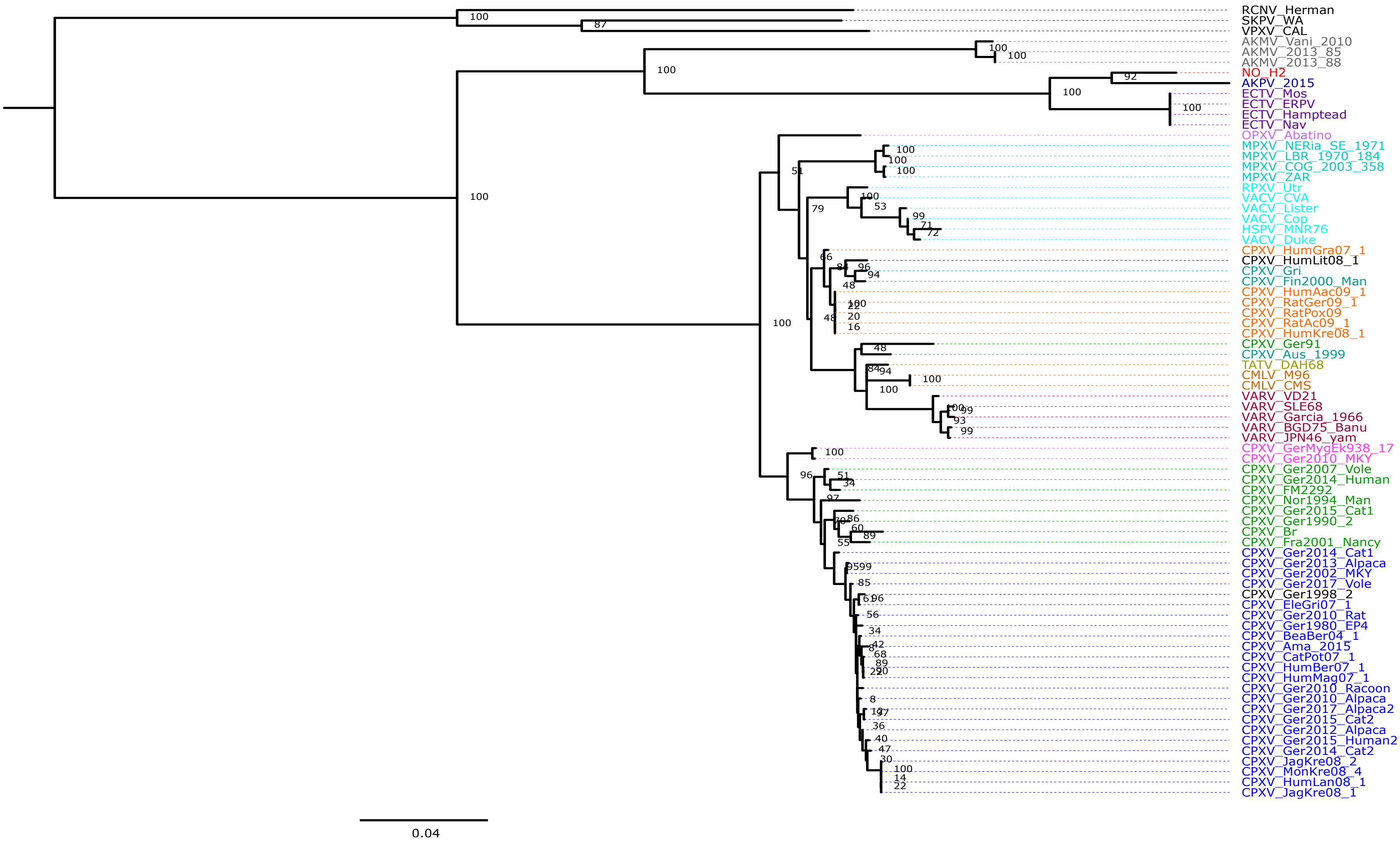

Supplementary Figure S7. Maximum-Likelihood phylogenetic tree based on the putative recombinant region 3 between the parental AKPV and CPXV (potential recombinant event 3). Bootstrap values were determined from 1000 replica sampling. Clades are identified with colors.

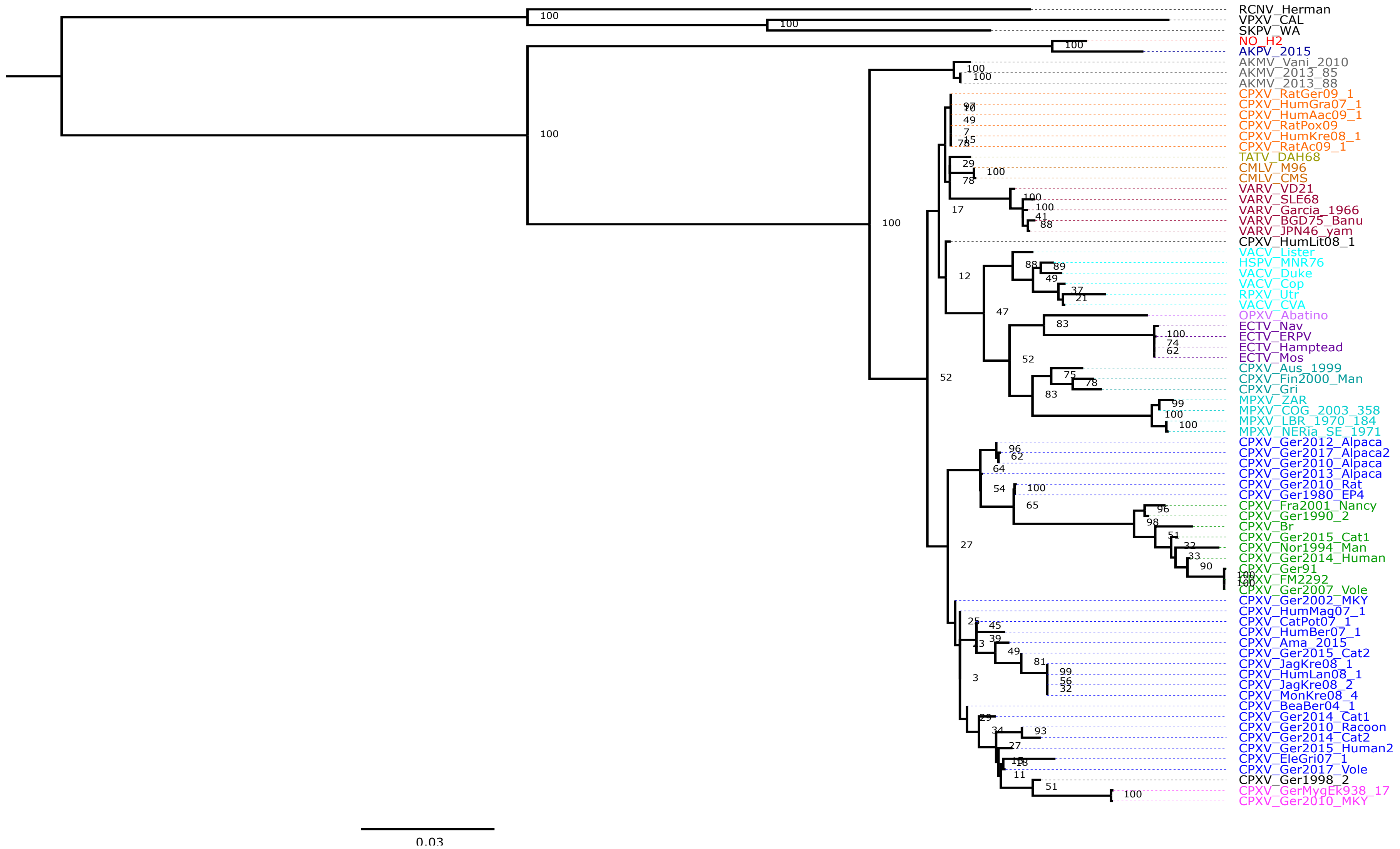

Supplementary Figure S8. Maximum-Likelihood phylogenetic tree based on the putative recombinant region 4 between the parental AKPV and CPXV (potential recombinant event 4). Bootstrap values were determined from 1000 replica sampling. Clades are identified with colors.

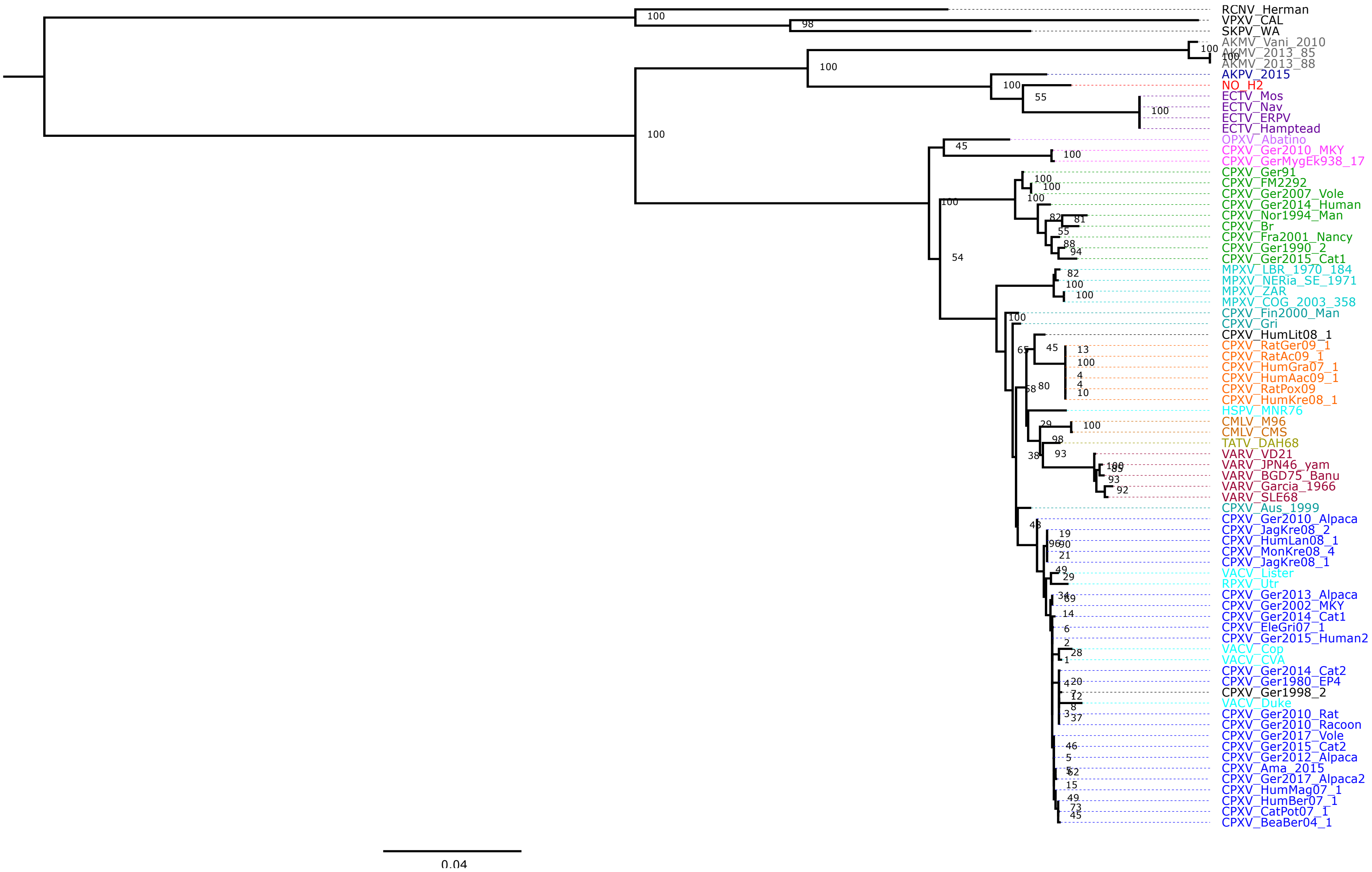

Supplementary Figure S9. Maximum-Likelihood phylogenetic tree based on the putative recombinant region 5 between the parental AKPV and CPXV (potential recombinant event 5). Bootstrap values were determined from 1000 replica sampling. Clades are identified with colors.

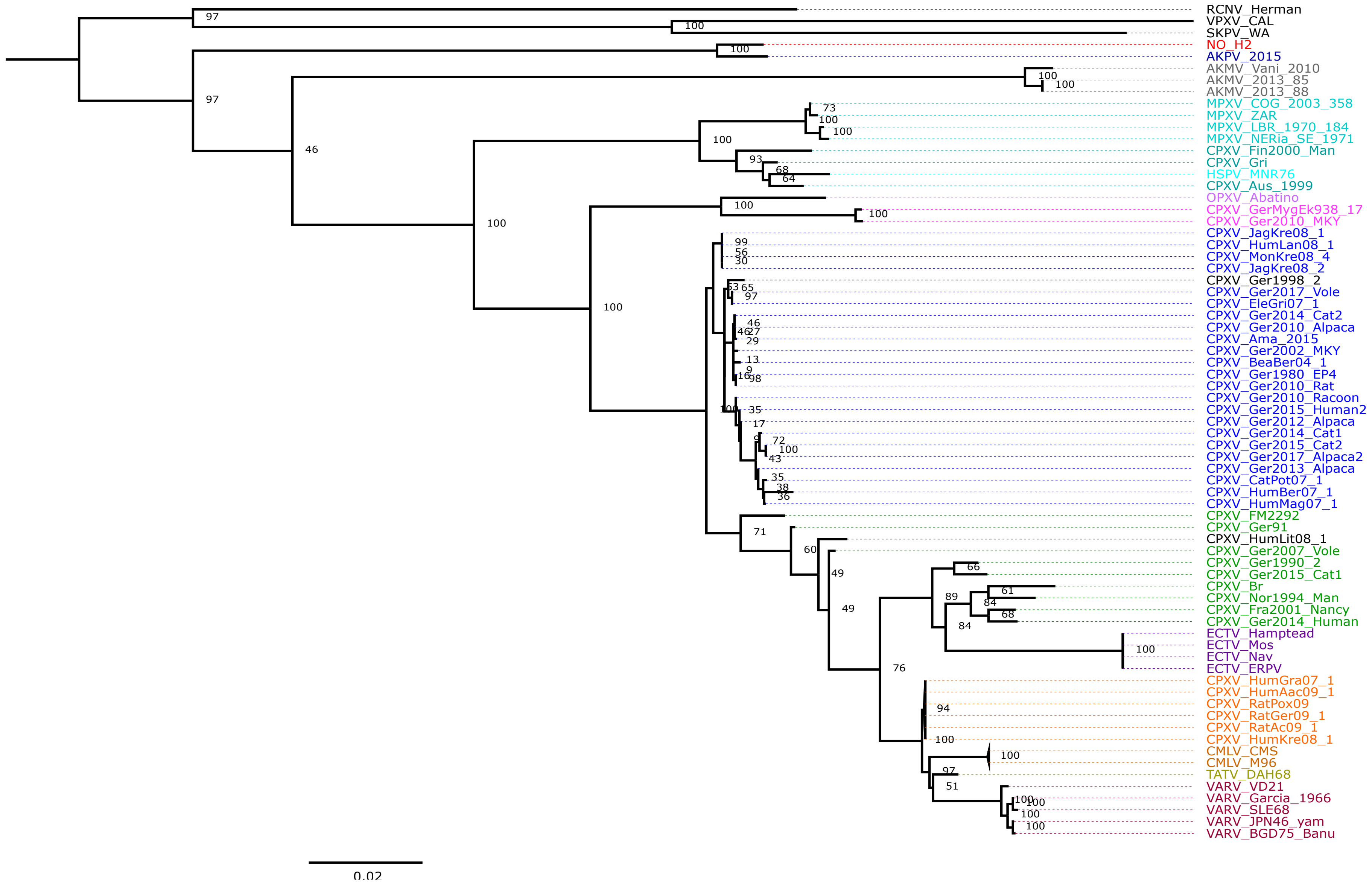

Supplementary Figure S10. Maximum-Likelihood phylogenetic tree based on the putative recombinant region 6 between the parental AKPV and CPXV (potential recombinant event 6). Bootstrap values were determined from 1000 replica sampling. Clades are identified with colors.

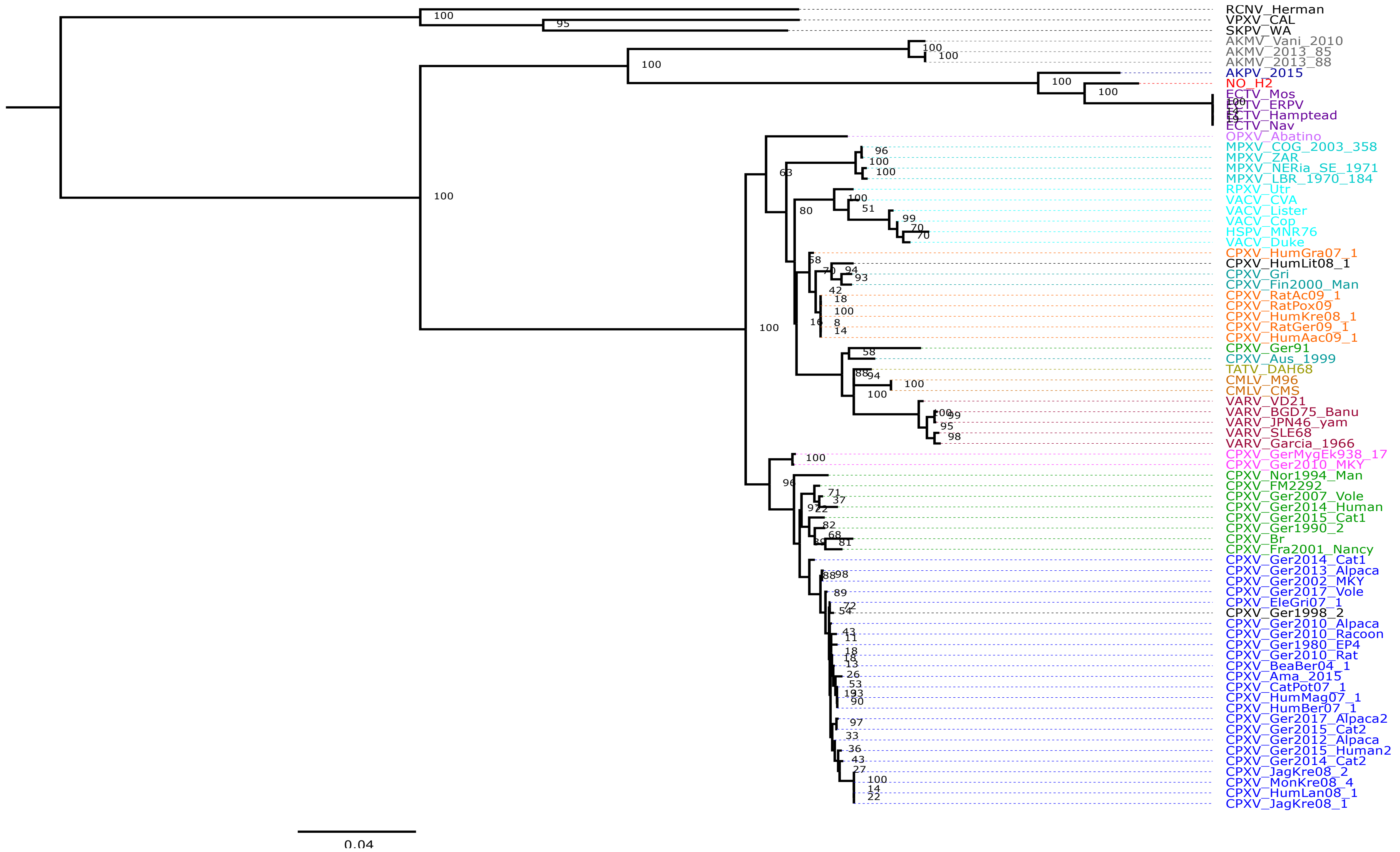

Supplementary Figure S11. Maximum-Likelihood phylogenetic tree based on the putative recombinant region 1 between the parental ECTV and CPXV (potential recombinant event 7). Bootstrap values were determined from 1000 replica sampling. Clades are identified with colors.

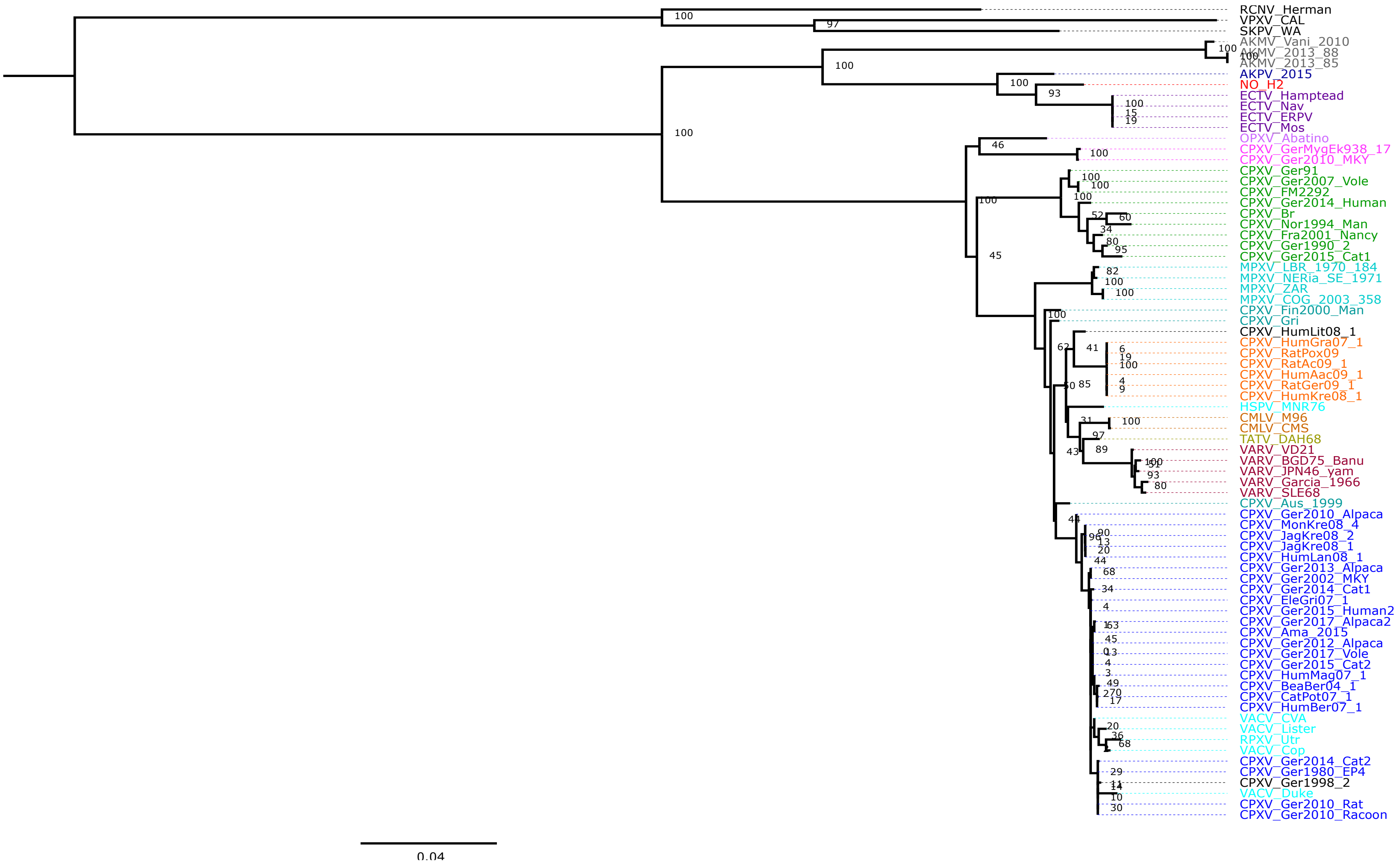

Supplementary Figure S12. Maximum-Likelihood phylogenetic tree based on the putative recombinant region 2 between the parental ECTV and CPXV (potential recombinant event 8). Bootstrap values were determined from 1000 replica sampling. Clades are identified with colors.

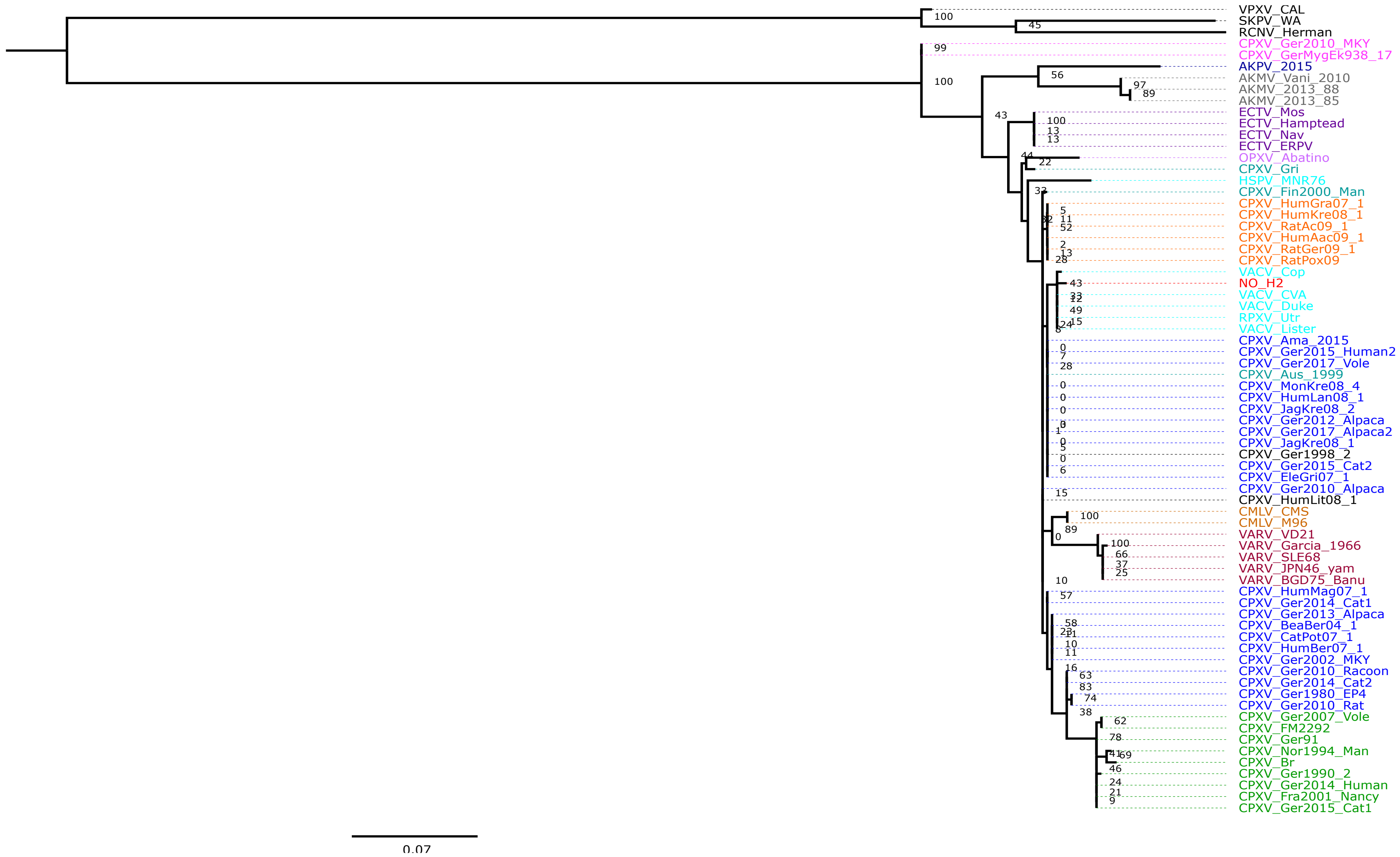

Supplementary Figure S13. Maximum-Likelihood phylogenetic tree based on the putative recombinant region between the parental VACV and CPXV (potential recombinant event 9). Bootstrap values were determined from 1000 replica sampling. Clades are identified with colors.

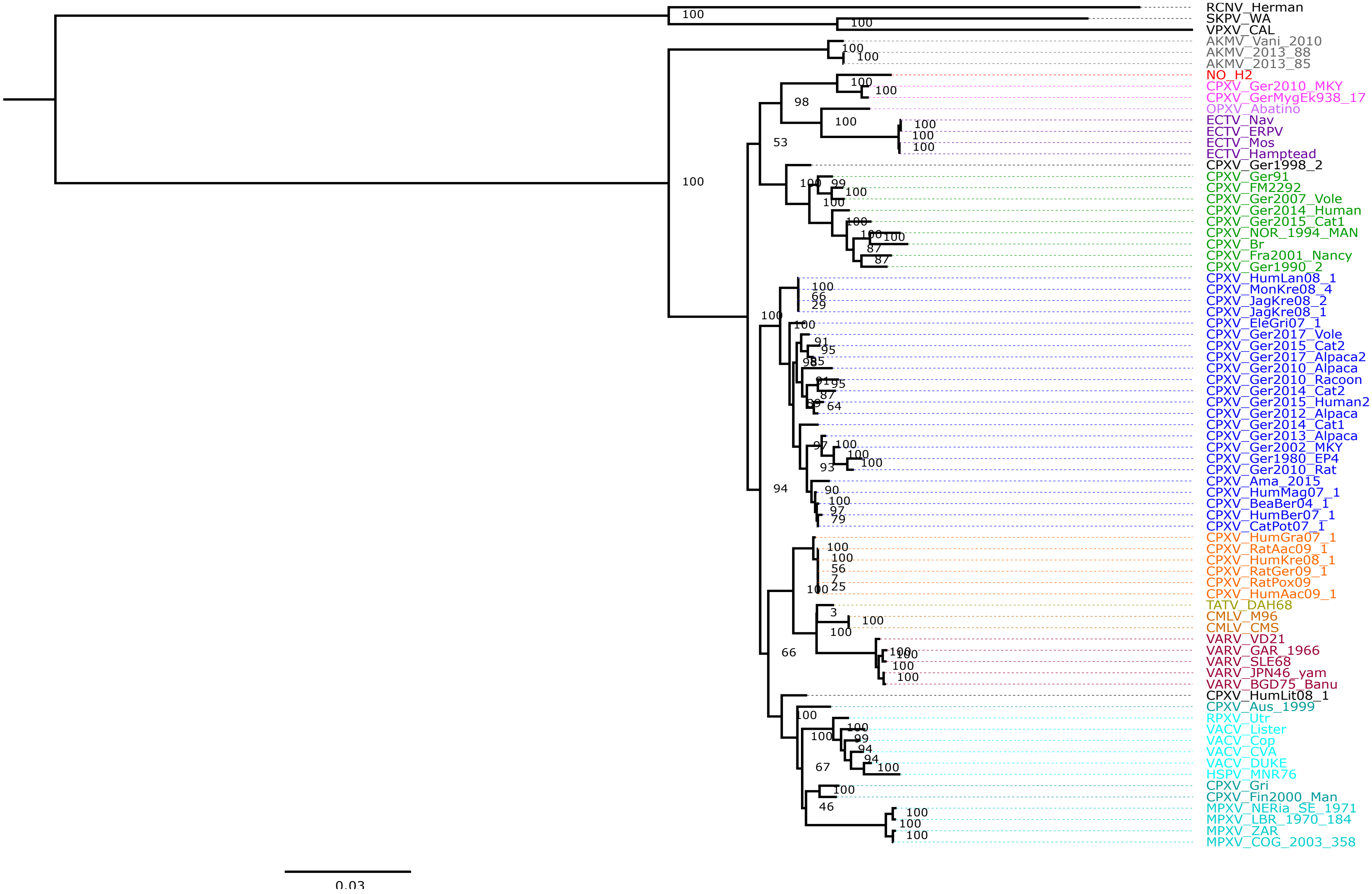

Supplementary Figure S14. Maximum-Likelihood phylogenetic tree based on 75 OPXV whole genomes. Bootstrap values were determined from 1000 replica sampling. Clades are identified with colors.

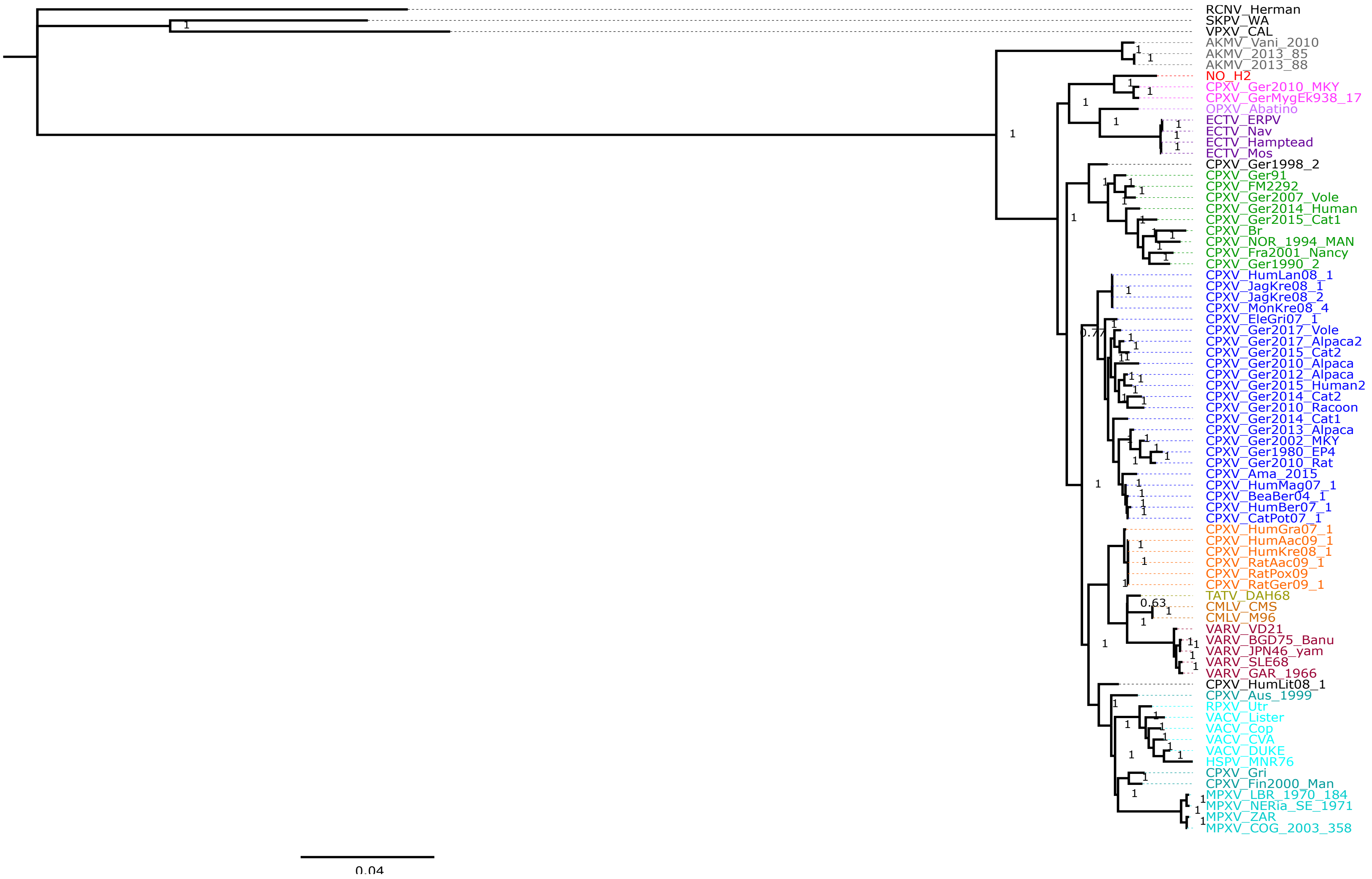

Supplementary Figure S15. Bayesian Inference phylogenetic tree based on 75 OPXV whole genomes. Clades are identified with colors.

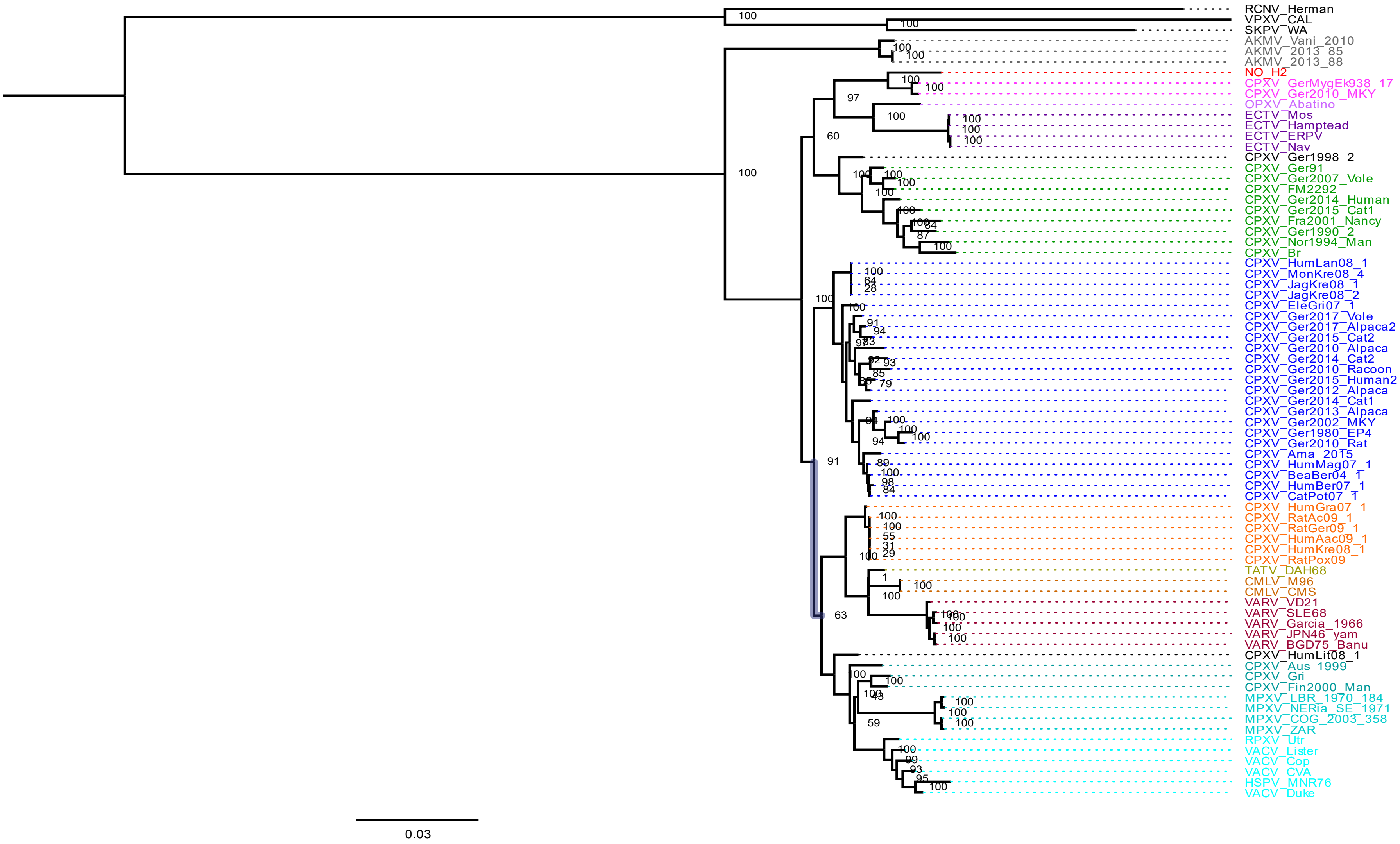

Supplementary Figure S16. Maximum-Likelihood phylogenetic tree based on 75 OPXV core genomes. Bootstrap values were determined from 1000 replica sampling. Clades are identified with colors.

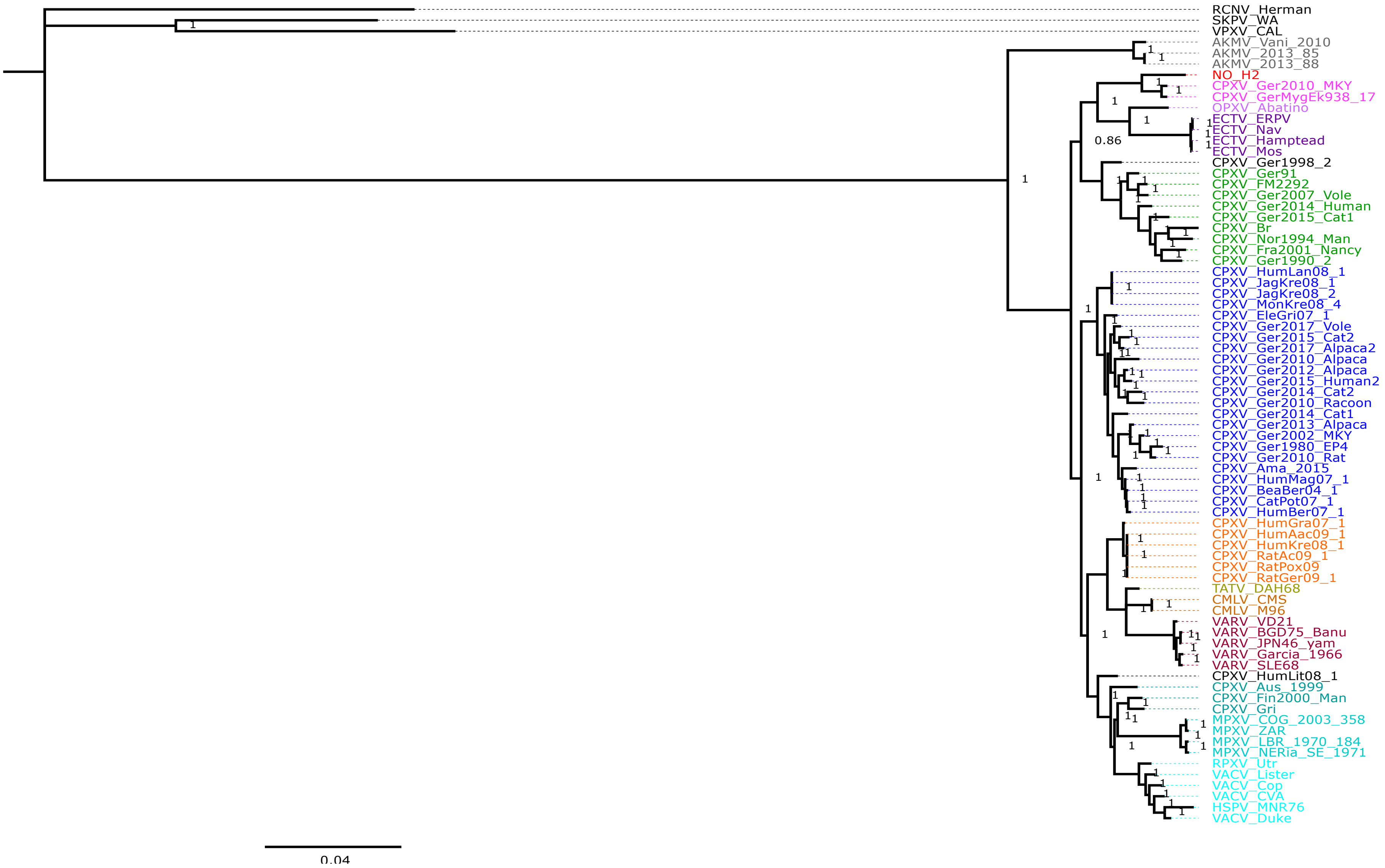

Supplementary Figure S17. Bayesian Inference phylogenetic tree based on 75 OPXV core genomes. Clades are identified with colors.

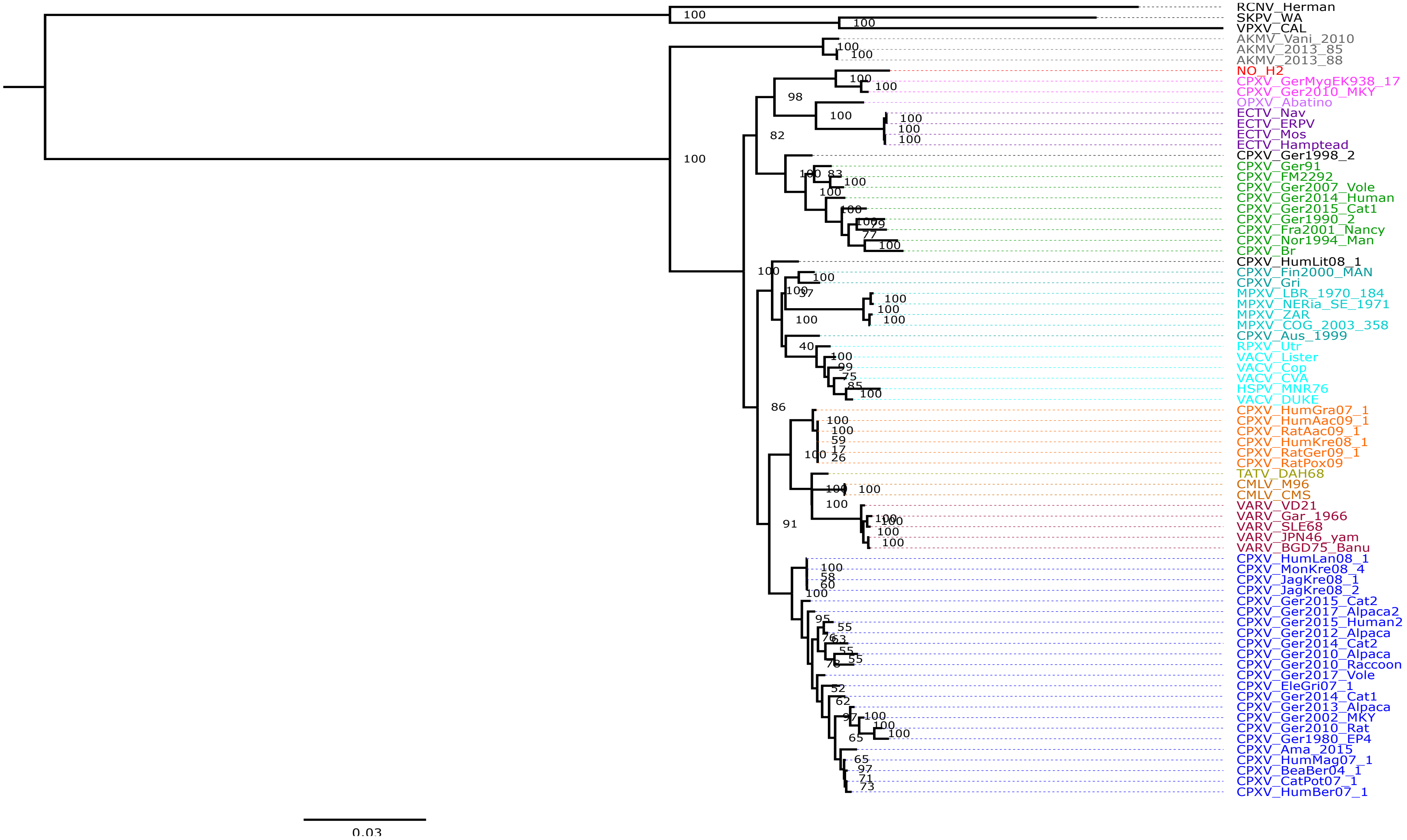

Supplementary Figure S18. Maximum-Likelihood phylogenetic tree based on 134 orthologous genes from 75 OPXV genomes. Bootstrap values were determined from 1000 replica sampling. Clades are identified with colors.

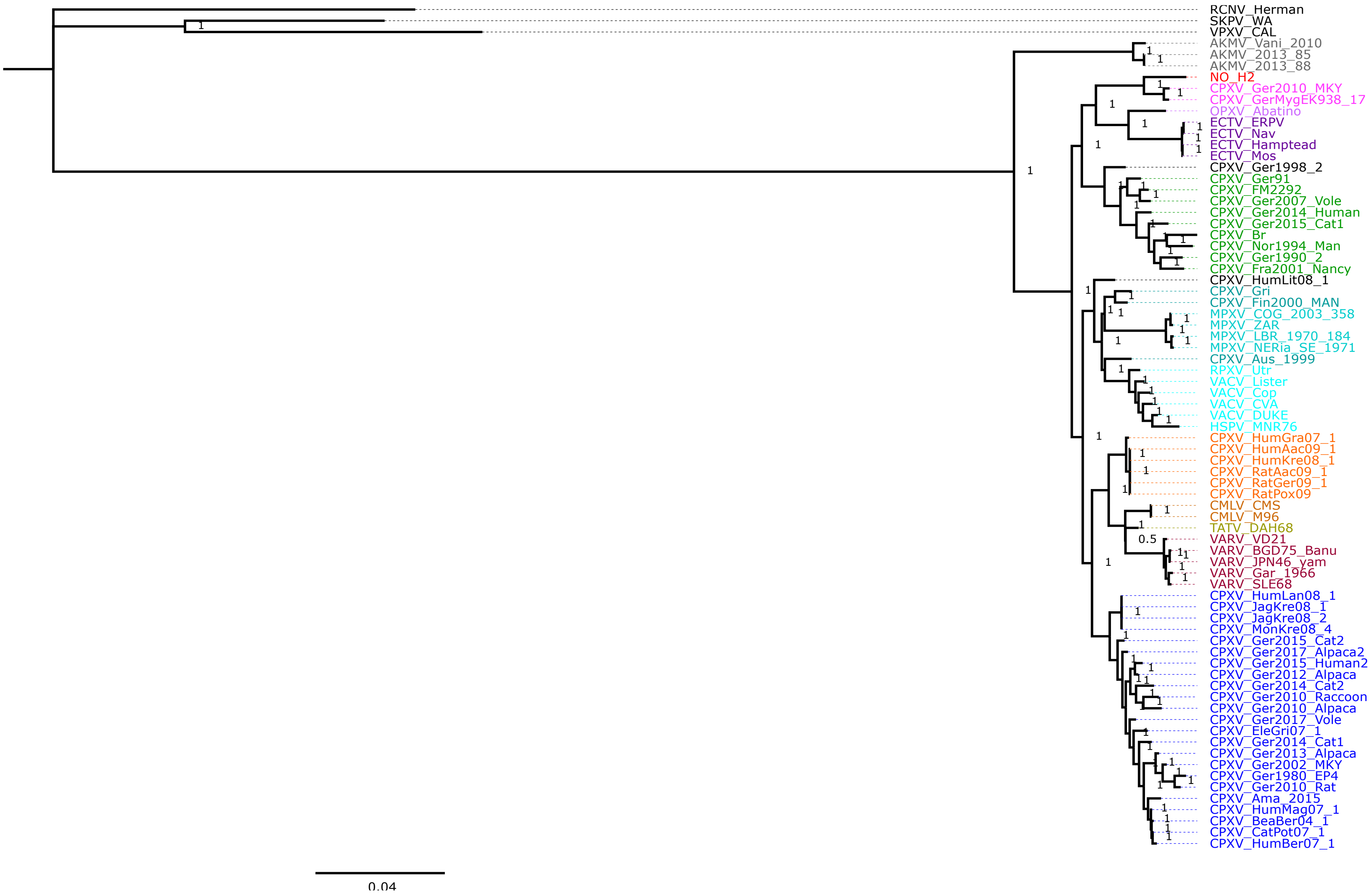

Supplementary Figure S19. Bayesian Inference phylogenetic tree based on 134 orthologous genes from 75 OPXV genomes. Clades are identified with colors.
